# A Quantum-Inspired Residual Self-Attention Network for Multimodal Sentiment Analysis

**DOI:** 10.3390/e28091014

**Published:** 2026-09-11

**Authors:** Yupeng Liu, Xianjie Feng, Yewang Zhong

**Affiliations:** Computer Science and Technology School, Harbin University of Science and Technology, Harbin 150000, China; 2025100810j4223@mails.cuc.edu.cn (X.F.); 2520410160@stu.hrbust.edu.cn (Y.Z.)

**Keywords:** quantum-inspired residual self-attention, complex-valued representations, multimodal fusion, feature selection

## Abstract

Multimodal sentiment analysis is challenging because textual, visual, and acoustic evidence is heterogeneous and oft en weakly aligned. Here, we present QRSAN, a quantum-inspired residual self-attention network that integrates an LSTM text encoder, modality-specific multilayer perceptrons for visual and acoustic inputs, normalized complex-valued encoding, a TFN-derived interaction expansion, real-valued residual self-attention, and classical projection-score mapping. QRSAN runs entirely on classical hardware and does not perform physical quantum computation. Across CMU-MOSI, CMU-MOSEI, and IEMOCAP, QRSAN was evaluated using a common protocol. It achieved the highest numerical mean ACC and Binary_F1 among the evaluated models on CMU-MOSI, whereas its IEMOCAP label-wise accuracy was below that of EF-LSTM. These findings support the utility of combining constrained complex-valued representations with residual self-attention using the evaluated settings, without claiming universal state-of-the-art performance.

## 1. Introduction

**Figure 1:**
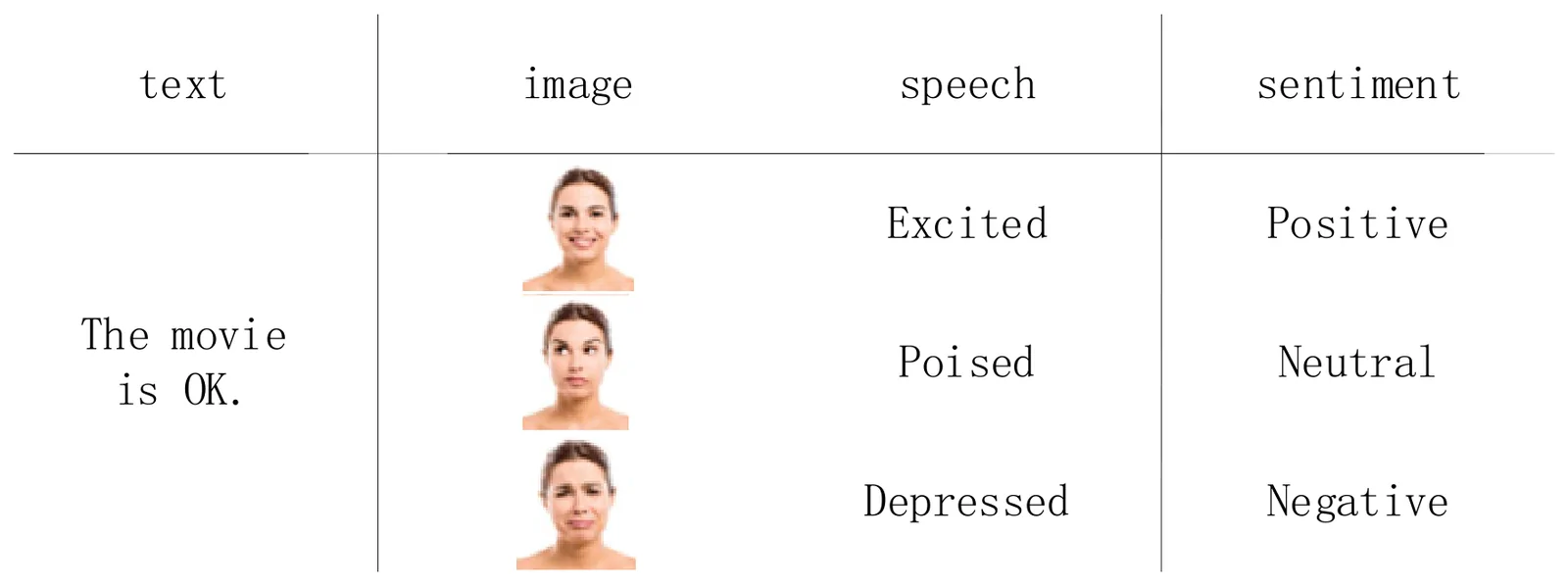
Multimodal sentiment example.

Multimodal sentiment analysis integrates textual, visual, and acoustic cues to infer a speaker’s affective state. For example, the sentence “The movie is OK.” may convey a positive, neutral, or negative attitude depending on the speaker’s facial expression and tone of voice (Figure 1).

Multimodal sentiment analysis exploits complementary information across modalities and can outperform unimodal approaches [1]. Fusion methods are commonly categorized as early, late, or hybrid strategies [2,3,4]. Early fusion combines features before model training, often through concatenation or parallel aggregation [5]. Late fusion combines modality-specific representations at intermediate or output layers, whereas hybrid strategies combine both approaches. Shared-subspace learning provides an additional route for reducing cross-modal heterogeneity [6].

QRSAN uses normalized complex-valued vectors and positive semidefinite matrices as classical representation learning tools. A TFN-derived interaction expansion combines these representations, which are subsequently processed by real-valued residual self-attention and mapped to sentiment scores through trainable projection directions. This formulation does not assume physical quantum-state preparation, entanglement, evolution, or measurement.

We examine how constrained complex-valued encoding, explicit higher-order interactions, and residual attention can be combined consistently for sentiment prediction. QRSAN is a quantum-inspired model implemented entirely on classical hardware. Its contributions are as follows.

(1) Mathematically constrained quantum-inspired modality encoding. We construct complex-valued modality representations from normalized amplitudes and non-negative mixture weights. The resulting matrices are Hermitian, positive semidefinite, and trace-normalized. This formulation is implemented on classical hardware and is not presented as physical quantum computation.
(2) TFN-derived interaction expansion with explicit normalization. Following the interaction expansion of TFN [7], we introduce a constant component into each modality representation to retain unimodal, bimodal, and trimodal interaction terms. Our contribution is not the tensor product expansion itself, but rather its mathematically defined block augmentation for normalized complex-valued representations.
(3) Real-valued residual self-attention for complex-valued fused features. Attention weights are computed from the real part of a complex-valued similarity matrix; therefore, Softmax is applied only to real-valued scores. The attention-transformed features are combined with the original fused representation through a residual connection.
(4) Classical projection score mapping and empirical evaluation. Classical projection score mapping transforms the residual attention output into real-valued projection scores for sentiment prediction. We evaluate the complete QRSAN architecture through modality-level and architecture-level comparisons on CMU-MOSI, CMU-MOSEI, and IEMOCAP.

## 2. Related Work

### 2.1. Multimodal Sentiment Analysis

Text sentiment analysis can be categorized by modelling granularity into document-level, sentence-level, and aspect-level approaches. Early work focused mainly on document-level [8,9,10] and sentence-level analysis [11,12,13]. Document-level approaches derive overall polarity from lexical or discourse cues. Chardon et al. [8] showed that discourse structure information annotated using Segmented Discourse Representation Theory [14] can be used to estimate overall polarity and contribution values in rating systems. Mittal et al. [9] developed rules for negation and discourse relation processing and aggregated word-level polarity values to determine overall sentiment. Märkle-Huß et al. [10] decomposed documents into elementary discourse units using rhetorical structure theory and organized them into binary trees [15]. Sentence-level methods use supervised or representation-learning approaches to classify sentiment. Liu et al. [11] combined general language and modal analysis features, whereas Fu et al. [12] used a semi-supervised phrase recursive autoencoder and bidirectional training classifier. Hayashi and Fujita [13] used word embeddings to estimate sentence polarity. More recent work has increasingly focused on aspect-level sentiment analysis [16,17], which assigns sentiment values to aspects mentioned in a document. Salas-Zárate et al. [16] proposed an ontology-based representation for knowledge sharing, and Xue and Li [17] introduced gated convolutional neural networks to model aspect and sentiment information.

Image sentiment analysis is more challenging than text sentiment analysis because it must relate visual content to affective meaning. Early work relied mainly on associations between low-level image features and sentiment. Machajdik and Hanbury [18] combined low-level features to represent affective image content. Subsequent studies incorporated semantic features. Feature-pyramid architectures have also supported multiscale visual representation learning [19]. Cao et al. [20] proposed the Visual Sentiment Topic Model, which aggregates images around a topic to improve representation learning. You et al. [21] used attention to identify emotion-related local regions, whereas Yuan et al. [22] introduced “Sentribute” as an intermediate attribute for visual-emotion classification. Gajarla and Gupta [23] evaluated the generalization of ResNet-50 for image-emotion categories on Flickr. Yang et al. [24] proposed a region-of-interest framework that identifies candidate objects, estimates emotion scores and perceived region importance, and combines these scores to identify affective regions adaptively.

Speech emotion recognition commonly extracts affective information from acoustic signals through feature extraction, fusion, and classification. Huang et al. [25] proposed a semi-supervised CNN to learn emotional features from labeled and unlabeled data. Lim et al. [26] combined convolutional and recurrent neural networks without relying on handcrafted features. Mirsamadi et al. [27] used local attention to identify salient regions of speech signals for emotion prediction. Liu et al. [28] developed an improved brain emotional learning model with a genetic algorithm for weight updating. Sajjad and Kwon [29] proposed similarity-measure clustering and key-sequence extraction to reduce computational complexity. Chen et al. [30] introduced masked speech denoising pretraining to predict pseudo-labels for masked regions.

Multimodal sentiment analysis [31,32,33,34] exploits complementarity and interactions among modalities. Yu et al. [31] combined text-only entity-span detection with a unified multimodal transformer. Sun et al. [32] used an LSTM and self-attention to model temporal feature dynamics before late fusion. He et al. [33] proposed multimodal temporal attention to model cross-modal temporal influences and adaptive modality balancing. Yu et al. [34] developed a multi-task architecture for coarse-to-fine image-target matching and target-sentiment classification. Attention-based neural fusion has also been applied directly to multimodal sentiment analysis [35]. Interpretability-oriented multimodal routing has also been proposed [36].

### 2.2. Quantum and Quantum-Inspired Sentiment Analysis

Quantum-related sentiment analysis methods can be broadly divided into physical quantum-computing approaches and quantum-inspired approaches implemented on classical computers [1]. Related quantum machine learning studies include neural network approaches to quantum dynamics, near-term quantum classifiers, and hybrid quantum LSTM architectures [37,38,39]. Zhang et al. [40] constructed sentiment lexicons using a quantum language model and used quantum relative entropy to measure similarity between lexicons and target objects. Zhang et al. [41] proposed a quantum-inspired interactive network that combines a CNN with density-matrix representations to model correlations among words within utterances. For multimodal analysis, Zhang et al. [42] introduced quantum-inspired multimodal representation (QMR) and quantum interference-inspired multimodal decision fusion (QIMF). QMR maps image and textual vocabularies into vector spaces and density-matrix representations, whereas QIMF addresses conflicts among modality-level decisions. Non-classical correlations in decision-fused multimodal documents have also been investigated using Bell inequality-based analysis [43]. Li et al. [44] proposed a quantum-like multimodal network for conversational sentiment analysis, combining interference-inspired decision fusion with a measurement-inspired influence model for adjacent utterances. Gkoumas et al. [45] represented modality features with complex-valued vectors and fused textual, visual, and acoustic information through an entanglement-inspired formulation.

The interaction expansion in QRSAN is derived from the classical Tensor Fusion Network (TFN) [7] and is not claimed as a new fusion operator. Unlike QMR and QIMF, which focus on quantum-inspired decision interference and measurement, QRSAN combines normalized complex-valued modality representations with residual self-attention. Unlike quantum-like conversational models, it focuses on utterance-level multimodal sentiment representations. QRSAN also differs from entanglement-driven fusion and QMF by applying Softmax only to a real-valued similarity matrix and by combining the attention-transformed representation with the original fused representation through a residual connection.

QRSAN therefore combines constrained complex-valued encoding, a TFN-derived interaction expansion, real-valued residual self-attention, and classical projection score mapping. It does not claim to introduce tensor-product fusion itself.

## 3. Model Architecture

**Figure 2:**
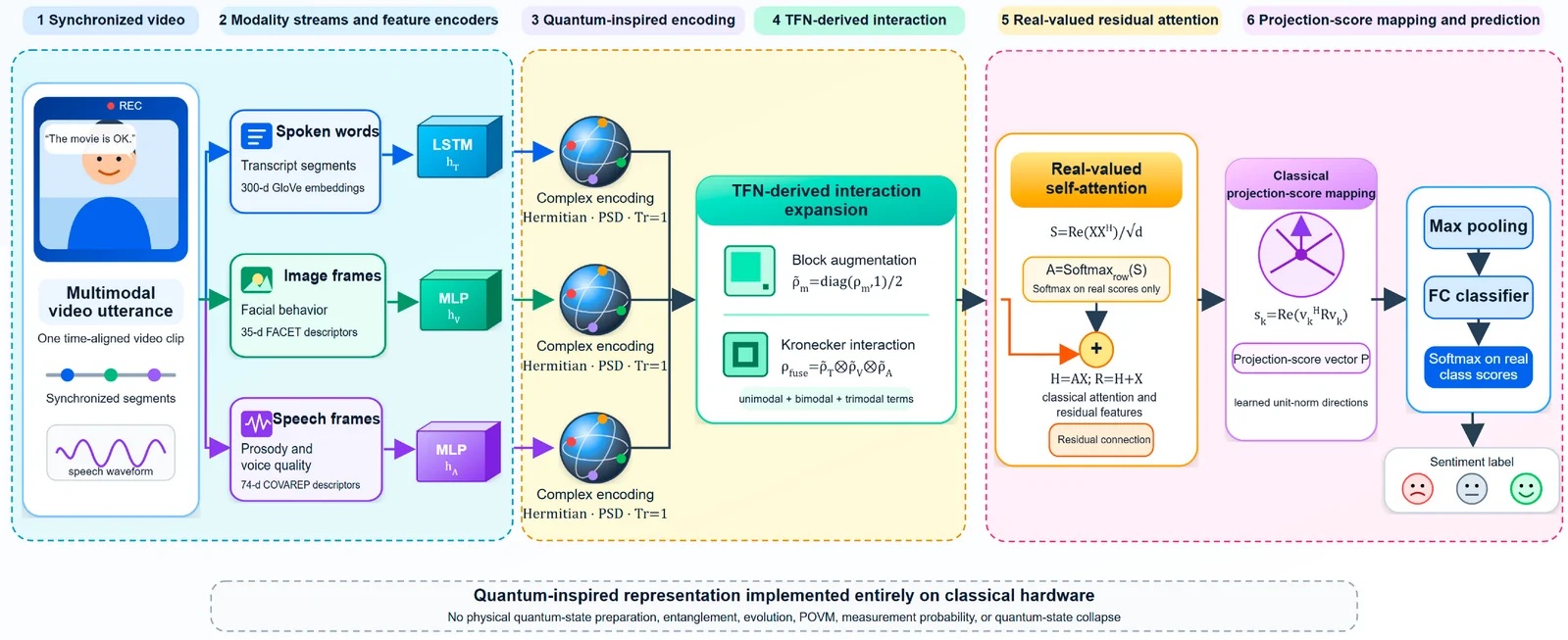
Architecture of the quantum-inspired QRSAN model.

QRSAN is implemented entirely on classical hardware and does not involve physical quantum computation. In this article, terms such as “quantum state”, “Hilbert space”, “tensor product”, and “measurement” denote mathematical representations or operations inspired by quantum theory rather than physical quantum processes. The overall architecture is shown in Figure 2.

QRSAN comprises four stages. First, an LSTM encodes text, whereas modality-specific multilayer perceptrons encode visual and acoustic sequences. Second, each modality is mapped to a normalized complex-valued representation, from which a Hermitian, positive semidefinite, and trace-normalized matrix is constructed. Third, the modality matrices are augmented and combined through a TFN-derived tensor-product interaction expansion. The joint representation is processed by a classical residual self-attention module whose weights are computed only from real-valued similarity scores. Finally, trainable complex projection kernels, initialized with unit Euclidean norm, map the residual output to sentiment scores.

### 3.1. Single Modality Representation

The LSTM network is used to capture dependencies among text fragments within a sentence. Formally, given an input text fragment, the previous cell state, and the previous hidden state, the current cell and hidden states are defined as follows:

(1) $$i_{k}=\sigma (W_{i}^{u}\times u_{k}^{t}+W_{i}^{h}\times h_{k-1}+b_{i})$$

(2) $$f_{k}=\sigma (W_{f}^{u}\times u_{k}^{t}+W_{f}^{h}\times h_{k-1}+b_{f})$$

(3) $$o_{k}=\sigma (W_{o}^{u}\times u_{k}^{t}+W_{o}^{h}\times h_{k-1}+b_{o})$$

(4) $${\hat{c}}_{k}=\tanh(W_{c}^{u}\times u_{k}^{t}+W_{c}^{h}\times h_{k-1}+b_{c})$$

(5) $$c_{k}=f_{k}\circ c_{k-1}+i_{k}\circ {\hat{c}}_{k}$$

(6) $$h_{k}=o_{k}\circ \tanh(c_{k})$$

In Equations (1)–(6), $i_{t}$, $f_{t}$, and $o_{t}$ denote the input, forget, and output gates, respectively; σ is the sigmoid function; W and b denote trainable weights and biases, respectively; and ⊙ denotes element-wise multiplication. The hidden state $h_{t}$ is used as the text representation.

Modality-specific MLPs encode the visual and acoustic inputs. These descriptors are already aligned with textual segments, and an MLP maps each segment to the shared representation dimension. Given a visual or acoustic feature segment $x^{m}$, the hidden activation and output representation are computed as follows:

(7) $$q_{j}^{m}=\sum_{i=0}^{M_{in}}w_{ij}^{m}u_{j}^{m}$$

(8) $$a_{j}^{m}=Sof\max(q_{j}^{m})$$

(9) $$x_{jk}^{m}=a_{j}^{m}$$

(10) $$h_{k}^{m}=\frac{\exp(\sum_{j=1}^{M_{hid}}w_{jk}^{m}x_{jk}^{m})}{\sum_{l=1}^{M_{out}}\exp(\sum_{j=1}^{M_{hid}}w_{jl}^{m}x_{jl}^{m})},k=1,\cdots ,M_{out}$$

Here, i, j, and k index the input components, hidden units, and output units, respectively, $M_{in}$, $M_{hid}$, and $M_{out}$ denote the corresponding dimensions, q is the weighted sum of the inputs, a is the neuron activation, x is the output-layer activation, and $h_{m}$ is the final MLP representation for modality m, where m∈{V,A} denotes the visual or acoustic modality.

We used word-aligned feature files distributed through the CMU Multimodal SDK and the Multimodal Transformer preprocessing pipeline. Text tokens were represented by 300-dimensional GloVe vectors and encoded sequentially by the text LSTM. Visual inputs comprised 35-dimensional FACET facial behavior descriptors, whereas acoustic inputs comprised 74-dimensional COVAREP descriptors of prosody and voice quality. Observations within each word interval were aligned with the corresponding token, and sequences were zero-padded to a fixed length. The implementation directly loads these pre-extracted arrays and does not train an image backbone or an end-to-end speech encoder. NaN and infinite values were replaced with zero before training.

### 3.2. Quantum-Inspired Encoding of Individual Modalities

For modality m∈{T,V,A}, each modality-specific feature segment is mapped to a normalized complex-valued vector $|h_{k}^{m}\rangle$ in the modality-specific feature space $H_{m}$. This vector is a mathematical feature representation implemented on classical hardware and is not a physically prepared quantum state. Let $K_{m}$ denote the number of feature segments and $d_{m}$ denote the dimension of the corresponding complex feature space. The k-th feature segment, where $k=1,2,\cdots ,K_{m}$, is encoded as follows:

(11) $$|h_{k}^{m}\rangle =\sum_{i=1}^{d_{m}}r_{ki}e^{i\theta_{ki}^{m}}|e_{i}^{m}\rangle ,m\in \{T,V,A\},k=1,2,\cdots ,K_{m}$$

Here, i denotes the imaginary unit, ${\left\{|e_{i}^{m}\rangle \right\}}_{i=1}^{d_{m}}$ is an orthonormal basis of the $d_{m}$-dimensional complex feature space, $r_{ki}^{m}\geq 0$ is the amplitude associated with the i-th basis component of the k-th segment, and $\theta_{ki}^{m}\in [-\pi ,\pi ]$ is its phase. The amplitudes are normalized according to

$$\sum_{i=0}^{d_{m}}{\left(r_{ki}^{m}\right)}^{2}=1$$

So that $\langle h_{k}^{m}|h_{k}^{m}\rangle =1$

The modality-level matrix is constructed as a convex mixture of the normalized segment projectors:

(12) $$\rho^{m}=\sum_{k=1}^{K_{m}}\lambda_{k}^{m}|h_{k}^{m}\rangle \langle h_{k}^{m}|$$

The mixture coefficients are computed from real-valued trainable scores $s_{k}^{m}$ using

$$\lambda_{k}^{m}=\frac{\exp\left(s_{k}^{m}\right)}{\sum_{l=1}^{K_{m}}\exp\left(s_{l}^{m}\right)}$$

Consequently, $\lambda_{k}^{m}\geq 0$ and $\sum_{k=1}^{K_{m}}\lambda_{k}^{m}=1$. Each projector $|h_{k}^{m}\rangle \langle h_{k}^{m}|$ is Hermitian and positive semidefinite. Hence, $\rho^{m}$ is Hermitian and positive semidefinite, and

$$\mathrm{Tr}\left(\rho^{m}\right)=\sum_{k=1}^{K_{m}}\lambda_{k}^{m}\mathrm{Tr}\left(|h_{k}^{m}\rangle \langle h_{k}^{m}|\right)=\sum_{k=1}^{K_{m}}\lambda_{k}^{m}=1$$

Thus, each modality-specific representation is Hermitian, positive semidefinite, and trace-normalized. These properties follow from amplitude normalization and non-negative, normalized mixture weights. The representation is constructed and processed entirely on classical hardware and serves only as a quantum-inspired mathematical constraint for representation learning; it is not claimed to be a physically prepared quantum state.

### 3.3. Quantum-Inspired Multimodal Fusion

Within the quantum-inspired formulation, textual, visual, and acoustic representations are combined to form a joint multimodal representation. Each modality is represented by a normalized complex-valued matrix derived from encoded features. These matrices are classical mathematical representations and do not correspond to physically prepared quantum states.

Following the interaction expansion of the Tensor Fusion Network (TFN) [45], we introduce a constant component into each modality-specific matrix. This augmentation retains unimodal and bimodal interactions alongside the trimodal interaction. Because each representation is square, the constant is introduced by block-diagonal augmentation rather than by directly appending a scalar.

Because $\rho^{m}$ is a square matrix, directly appending a scalar to it would result in an ambiguously defined matrix. Therefore, the constant component is introduced by adding one row and one column to the original matrix:

(13) $${\bar{\rho }}^{m}=\frac{1}{2}\left[\begin{array}{ll} \rho^{m} & 0\\ 0^{†} & 1 \end{array}\right],m\in \{T,V,A\}.$$

Here, $0\in C^{d_{m}\times 1}$, and therefore ${\bar{\rho }}^{m}\in C^{\left(d_{m}+1\right)\times \left(d_{m}+1\right)}$. We next verify that the augmented representation preserves Hermiticity, positive semidefiniteness, and unit trace.

First, since $\rho^{m}$ is Hermitian, i.e., ${\left(\rho^{m}\right)}^{H}=\rho^{m}$, we have

$${\left({\bar{\rho }}^{m}\right)}^{H}=\frac{1}{2}\left[\begin{array}{l} {\left(\rho^{m}\right)}^{H}\\ 0^{†} \end{array}\right.\left.\begin{array}{l} 0\\ 1 \end{array}\right]={\bar{\rho }}^{m}$$

Thus, ${\bar{\rho }}^{m}$ is Hermitian. Second, for any vector $z={\left[u^{T},a\right]}^{T}\in C^{\left(d_{m}+1\right)}$, where $u\in C^{d_{m}}$ and a∈C, we obtain

$$z^{H}{\bar{\rho }}^{m}z=\frac{1}{2}(u^{H}\rho^{m}u+{\left|a\right|}^{2})\geq 0$$

Because $\rho^{m}$ is positive semidefinite, $u^{H}\rho^{m}u\geq 0$ for every $u\in C^{d_{m}}$. Therefore, ${\bar{\rho }}^{m}$ is also positive semidefinite. Finally,

$$\mathrm{Tr}({\bar{\rho }}^{m})=\frac{1}{2}\left[\mathrm{Tr}(\rho^{m})+1\right]=\frac{1}{2}[1+1]=1.$$

Therefore, the block-diagonal augmentation in Equation (13) preserves Hermiticity, positive semidefiniteness, and unit trace. The augmented representation is used as a classical quantum-inspired feature representation and is not interpreted as a physically prepared quantum state.

The three augmented modality representations are subsequently combined through the Kronecker product:

(14) $$\rho_{mm}={\bar{\rho }}^{T}⊗{\bar{\rho }}^{V}⊗{\bar{\rho }}^{A}.$$

To distinguish the dimensionality before and after block-diagonal augmentation, we denote the unaugmented trimodal core interaction dimension by $D_{core}=d_{T}d_{V}d_{A}$ and the dimension of the augmented joint representation by $D=(d_{T}+1)(d_{V}+1)(d_{A}+1)$. In the reported configuration, $d_{T}=d_{V}=d_{A}=10$; hence, $D_{core}=10\times 10\times 10=1000$, whereas $D_{aug}=11\times 11\times 11=1331$. The additional 331 coordinates arise from the constant components and retain lower-order interaction terms in the TFN-derived expansion. All subsequent attention and projection operations act on the augmented representation of dimension $D_{aug}=1331$.

The added constant components retain lower-order interaction terms and ensure that the complete tensor-product representation remains non-zero when one modality contributes a zero or near-zero feature value.

Because each augmented modality representation ${\bar{\rho }}^{m},m\in \{T,V,A\}$ is Hermitian, positive semidefinite, and trace-normalized, the joint representation in Equation (14) preserves these properties. Specifically,

$$\rho_{mm}^{H}={\left({\bar{\rho }}^{T}⊗{\bar{\rho }}^{V}⊗{\bar{\rho }}^{A}\right)}^{H}={\left({\bar{\rho }}^{T}\right)}^{H}⊗{\left({\bar{\rho }}^{V}\right)}^{H}⊗{\left({\bar{\rho }}^{A}\right)}^{H}=\rho_{mm}$$

Thus, $\rho_{mm}$ is Hermitian. In addition, the Kronecker product of positive semidefinite matrices is positive semidefinite. Since ${\bar{\rho }}^{T}\geq 0$, ${\bar{\rho }}^{V}\geq 0$, and ${\bar{\rho }}^{V}\geq 0$, it follows that $\rho_{mm}\geq 0$. Finally,

$$Tr(\rho_{mm})=Tr({\bar{\rho }}^{T})Tr({\bar{\rho }}^{V})Tr({\bar{\rho }}^{A})=1$$

Therefore, the fused representation is Hermitian, positive semidefinite, and trace-normalized before entering the residual self-attention module. The added constant components retain lower-order interaction terms and prevent the complete tensor-product representation from vanishing when one modality contributes a zero or near-zero feature value.

Block-Wise Interaction Expansion and Complexity.

Let $n_{m}=d_{m}+1$ for m∈{T,V,A}, and let $D=n_{T}n_{V}n_{A}$ denote the dimension of the augmented joint representation. The unaugmented trimodal core has dimension $D_{core}=d_{T}d_{V}d_{A}$. For the reported configuration, $D_{core}=1000$ and $D_{aug}=1331$. An explicit dense realization of $\rho_{mm}\in C^{\left(D\times D\right)}$ requires $O(D^{2})$ storage. The block-diagonal augmented matrix ${\bar{\rho }}^{m}$ contains only the $d_{m}\times d_{m}$ block $\rho^{m}$ and one scalar diagonal entry. Hence, it contains ${d_{m}}^{2}+1$ potentially nonzero entries rather than ${n_{m}}^{2}$ entries. In the implementation, the zero-valued off-diagonal blocks are not materialized. Instead, each augmented representation is represented by the pair $\left[\rho^{m},1\right]$ together with the factor 1/2, and only the nonzero interaction blocks are evaluated. This avoids storage and multiplication involving the zero off-diagonal blocks. However, this block-wise realization does not eliminate the high-order cost of subsequent dense operations once the joint D×D feature matrix is used by the attention module.

The augmented Kronecker product is a mathematically constrained, quantum-inspired feature-fusion operation. It is not interpreted as physical quantum entanglement or as a physically prepared multimodal quantum state.

**Figure 3:**
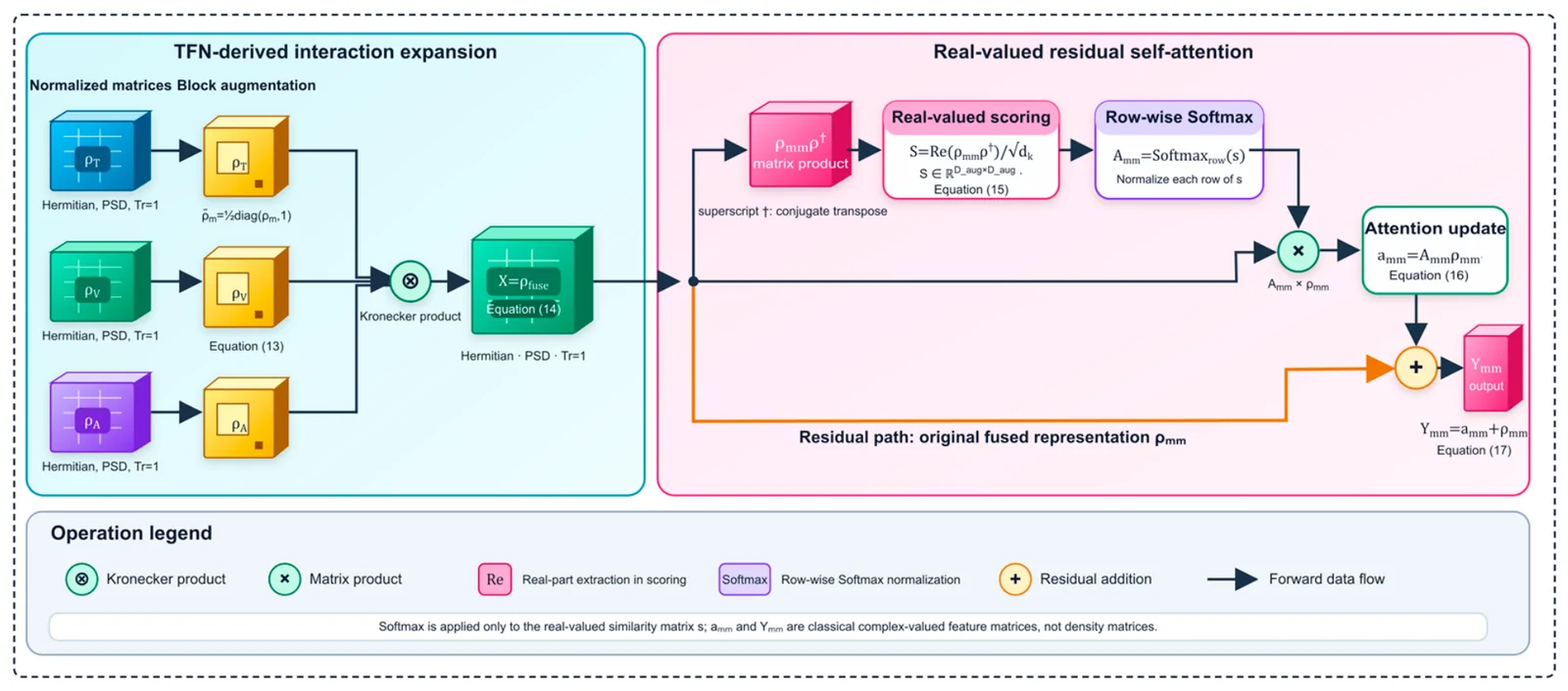
TFN-derived block-diagonal interaction expansion and real-valued residual self-attention in QRSAN. Each modality matrix is augmented with a constant diagonal component before the Kronecker product is computed. With contracted modality dimensions (10,10,10), the unaugmented trimodal core has $D_{core}=1000$ coordinates, whereas the augmented fused representation has $D_{aug}=1331$ coordinates. Row-wise Softmax is applied only to the real-valued similarity scores.

The joint representation is then processed by residual self-attention. The attention mechanism emphasizes features relevant to sentiment prediction, whereas the residual connection preserves information from the original fused representation. At this stage, the fused representation and attention outputs are treated as feature matrices rather than physically valid quantum density matrices. The attention and residual operations are therefore classical neural network operations, not physical quantum evolution. Figure 3 illustrates the procedure.

Coordinate Semantics and Row-Wise Attention. The rows and columns of $\rho_{mm}$ are indexed by the learned tensor-product coordinate system formed from the augmented textual, visual, and acoustic coordinates. More specifically, each index corresponds to one joint interaction coordinate in the product basis. The i-th row of $\rho_{mm}$ is the complex-valued feature profile associated with the i-th joint interaction coordinate, and the j-th column specifies its response along the j-th coordinate. These indices do not represent temporal positions, tokens, or physical quantum states. The joint multimodal representation is subsequently fed into a residual self-attention module. Since $\rho_{mm}$ takes complex values, the attention score matrix is converted into a real-valued matrix before Softmax normalization. The row-wise attention weights are computed via

(15) Amm=SoftmaxrowReρmmρmm†dk

where $\rho_{mm}^{†}$ denotes the conjugate transpose of $\rho_{mm}$, and Re(·) extracts the real component of the similarity matrix.

Let $D_{aug}$ denote the dimension of the augmented fused representation. Thus, $\rho_{mm}\in {\mathbb{C}}^{D_{aug}\times D_{aug}}$, Reρmmρmm†/Daug∈ℝDaug×Daug, and $A_{mm}=Soft\max_{row}\left(s\right)\in {\mathbb{R}}^{D_{aug}\times D_{aug}}$. For the reported configuration, $D_{aug}=1331$, and the scaling factor in Equation (15) is therefore $\sqrt{1331}$. Softmax is applied independently to each row of s, and every row of $A_{mm}$ sums to one.

The attention-aware updated representation is yielded by

(16) $$a_{mm}=A_{mm}\rho_{mm}$$

And the residual output is calculated as

(17) $$Y_{mm}=a_{mm}+\rho_{mm}$$

Thus, Softmax is not directly applied to a complex-valued fused representation. Instead, it is applied only to the real-valued similarity-score matrix Re(ρmmρmm†)/D in Equation (15).

After the fusion stage, $\rho_{mm}$ is used only as a complex-valued feature matrix in the residual self-attention module. Equations (15)–(17) are classical neural network operations: $A_{mm}$ is a real-valued row-normalized attention matrix, $a_{mm}=A_{mm}\rho_{mm}$ is the attention-aware feature matrix, and $Y_{mm}=a_{mm}+\rho_{mm}$ is the residual feature matrix. Since $A_{mm}$ is generally not symmetric, neither $a_{mm}$ nor $Y_{mm}$ is guaranteed to preserve Hermiticity, positive semidefiniteness, or unit trace.

Therefore, $a_{mm}$ and $Y_{mm}$ are treated exclusively as classical complex-valued neural features. They are not interpreted as density matrices, physical quantum states, quantum evolution, or physical quantum measurements.

Dense-Attention Complexity. For a dense $D_{aug}\times D_{aug}$ feature matrix, forming the score matrix in Equation (15) requires $O({D_{aug}}^{3})$ time and $O({D_{aug}}^{2})$ memory, whereas row-wise Softmax requires $O({D_{aug}}^{2})$ time. Computing $a_{mm}=A_{mm}\rho_{mm}$ in Equation (16) also requires $O({D_{aug}}^{3})$ time and $O({D_{aug}}^{2})$ memory. For K projection directions, the projection-score mapping in Equation (18) requires $O(K{D_{aug}}^{2})$ time when each quadratic form is evaluated directly. Thus, block-wise interaction expansion avoids computations involving structural zero blocks during augmentation, but the dense attention operations remain the dominant asymptotic cost.

### 3.4. Classical Projection-Score Mapping

The projection-score module processes the residual attention output for sentiment recognition. Because this output is a classical complex-valued feature matrix rather than a physical density matrix, the module computes real-valued discriminative projection scores rather than physical measurement probabilities.

For each trainable complex projection kernel of dimension D, the model computes the real-valued projection score using Equation (18). The projection scores from all learned kernels are collected in the projection-score vector P. Specifically,

(18) rk=Revk†Ymmvk=ReTrYmmvk†vk.

(19) $$P={\left[r_{1},r_{2},\cdots ,r_{k}\right]}^{T}.$$

The projection kernels and their associated rank-one matrices are not interpreted as elements of a physical POVM. Because the residual output is not generally Hermitian, positive semidefinite, or trace-normalized, each score can take a general real value and is used only as a discriminative feature.

The residual attention output is aggregated by max pooling and passed to the default MLP classifier. The final fully connected output layer of this MLP, as defined in Equation (20), produces real-valued class logits, to which Softmax in Equation (21) is applied. The sensitivity analysis presented later evaluates alternative pooling and classifier-head designs while keeping the remaining model components unchanged. These alternative heads do not redefine the architecture used for the main reported results. In the reported configuration, K=20 projection kernels are used.

Each projection kernel operates on the augmented fused representation, and therefore has $D_{aug}=1331$ complex entries, i.e., $v_{k}\in {\mathbb{C}}^{D_{aug}}$. The kernels are stored as a real-imaginary tensor of shape (20,1331,2). The unaugmented core interaction dimension is $D_{core}=1000$; it is reported only to distinguish the trimodal core coordinates from the complete augmented representation. Each projection kernel is normalized to unit Euclidean norm at initialization only. No post-update renormalization, unit-norm constraint, orthogonality penalty, or orthogonality constraint is applied during training; the kernels are therefore unconstrained trainable complex parameters rather than a normalized measurement basis. The resulting projection-score vector is max-pooled and passed to the default MLP classifier. For classification, the MLP outputs C real-valued logits, followed by Softmax and cross-entropy loss. For regression, the same feature extractor, projection-score mapping, and max-pooling operation are followed by a separate scalar output head trained with MAE loss. The empirical redundancy analysis of the learned kernels is reported in Section 4.5.

(20) $$s_{i}=W_{i}\max(P)+b_{i},i=1,2,\cdots ,C,$$

(21) $${\hat{y}}_{i}=\frac{\exp\left(s_{i}\right)}{\sum_{i=0}^{C}\exp\left(s_{i}\right)},i=1,2,\cdots ,C$$

Here, $W_{i}$ and $b_{i}$ denote the weight and bias associated with the i-th sentiment class, respectively; $s_{i}$ is the real-valued logit for class i; the predicted probability is defined for each class i; C denotes the number of sentiment classes; and max(•) denotes max pooling. Because Softmax is applied only to the real-valued class logits, the resulting probability vector is a conventional classifier probability distribution rather than a quantum measurement probability.

The projection-score module is therefore a classical projection-based feature-mapping operation. It does not represent physical quantum measurement, a POVM, or quantum state collapse.

### 3.5. Training Procedure

The trainable parameters of QRSAN include the LSTM and modality-specific MLP parameters, the parameters used to generate modality-specific amplitudes and phases, the mixture-weight scores, the trainable projection kernels, and the weights and biases of the final classifier. Hyperparameters were selected by grid search on the validation set. The same search space was applied independently to CMU-MOSEI, CMU-MOSI, and IEMOCAP. The search space comprised batch size {8, 16, 24, 32}; learning rate from $1\times 10^{-5}$ to $1\times 10^{-2}$; gradient-norm clipping threshold from 0.5 to 5.0; training epochs {5, 10, 15, 20, 25}; random seed {77, 78, 79, 80, 81}; hidden dimension {16, 32, 64, 128, 256}; and number of projection kernels {5, 10, 15, 20, 25, 30}. The final configuration used for the reported experiments was seed 77, batch size 32, 20 training epochs, RMSProp with a learning rate of 0.001, gradient-norm clipping at 1.0, hidden dimension 128, contracted modality dimensions (10, 10, 10), 20 projection kernels, and dropout 0.2. The selected configuration was identical across CMU-MOSEI, CMU-MOSI, and IEMOCAP. For each candidate configuration, the checkpoint with the lowest validation negative log-likelihood was selected. The test set was used only for final evaluation.

All experiments were conducted on a Linux host equipped with an Intel Xeon Platinum 8168 CPU (96 logical cores), 251.52 GiB RAM, and four NVIDIA GeForce RTX 3090 GPUs with 24 GiB memory each. The software environment comprised Python 3.10.20, PyTorch 2.5.1+cu124, and CUDA runtime 12.4. Unless otherwise stated, each run used a single GPU.

## 4. Experiments and Results

### 4.1. Datasets

Three multimodal sentiment and emotion datasets were used in this study, as summarized in Table 1: the Carnegie Mellon University Multimodal Opinion-Level Sentiment Intensity dataset (CMU-MOSI), the Carnegie Mellon University Multimodal Opinion Sentiment and Emotion Intensity dataset (CMU-MOSEI), and the Interactive Emotional Dyadic Motion Capture dataset (IEMOCAP). CMU-MOSI contains 2200 opinion video clips with binary, five-class, and seven-class sentiment labels [46]. The original CMU-MOSEI corpus contains more than 23,500 annotated utterances [47]. After alignment, preprocessing, and removal of unavailable or invalid samples, 22,777 utterances remained: 16,265 for training, 1869 for validation, and 4643 for testing. CMU-MOSEI was evaluated using binary, five-class, and seven-class sentiment labels. IEMOCAP contains approximately 12 h of audiovisual recordings with speech, facial motion capture, and transcripts [48]. The four emotion labels evaluated here were anger, happiness, sadness, and neutrality. The reported IEMOCAP experiments use a prepared instance-level feature pickle containing 2717 training, 798 validation, and 938 test instances (4453 in total). The original IEMOCAP corpus contains five dyadic sessions and ten speakers. In an accompanying upstream IEMOCAP representation that retains dialog identifiers, the source split uses Sessions 01–03 for training, Session 04 for validation, and Session 05 for testing; this source split contains no session or speaker-role overlap across partitions. However, the prepared feature pickle used for the reported experiments retains only labels, text, audio, and visual features and does not retain the original dialog identifiers, speaker identifiers, or session identifiers. Moreover, its utterance counts differ from those of the identifier-retaining source representation. Therefore, the correspondence between the prepared feature pickle and the source session split cannot be verified at the utterance level. The IEMOCAP results are reported under the supplied instance-level split. Because the prepared feature pickle lacks original utterance, speaker, and session identifiers, speaker/session disjointness cannot be verified at the utterance level.

For CMU-MOSI and CMU-MOSEI binary sentiment classification, labels greater than or equal to zero are assigned to the non-negative class, whereas labels below zero are assigned to the negative class. Zero-valued samples are retained and are not removed from binary evaluation. For ACC_5, labels and predictions are clipped to the interval [−2, 2] and rounded using the implementation’s numerical rounding rule. For ACC_7, labels and predictions are rounded to the original seven-level sentiment scale.

IEMOCAP is evaluated as four independent one-vs-rest binary emotion-label tasks over anger, happiness, sadness, and neutrality. For each utterance and emotion label, the model outputs two logits representing the negative and positive classes, respectively; the label-specific prediction is obtained by applying argmax to these two logits. Thus, the output has four binary label decisions per utterance rather than one mutually exclusive four-class decision. The primary IEMOCAP metric is micro-averaged label-wise accuracy, calculated as the number of correct binary label decisions divided by 4N, where N is the number of utterances. For the robustness analysis, balanced accuracy and macro-F1 are macro-averaged across the four one-vs-rest emotion-label tasks.

### 4.2. Comparison of Baseline Models

The following classical baseline systems were used for comparison with QRSAN.

(1) **Early-Fusion LSTM (EF-LSTM)**

EF-LSTM concatenates the textual, visual, and acoustic features at each time step and uses an LSTM to encode the resulting early-fusion sequence. The final hidden representation is used for prediction.

(2) **Late-Fusion LSTM (LF-LSTM)**

LF-LSTM first encodes the textual, visual, and acoustic sequences with separate LSTMs. Their final hidden representations are then concatenated for prediction.

(3) **Multi-Attention Recurrent Network (MARN)** [49]

MARN uses LSTM networks to learn hidden states for the three modalities at each time step. A multi-attention mechanism then fuses the modality representations and feeds the fused information back into the recurrent update. The fused representation and hidden states are subsequently combined to form a multimodal sentence representation for prediction.

(4) **Memory Fusion Network (MFN)** [50]

MFN augments modality-specific LSTMs for text, visual, and acoustic inputs with a memory component. The memory is updated from the learned LSTM representations, and the resulting memory unit is concatenated with the modality hidden states to form a multimodal representation for prediction.

(5) **Tensor Fusion Network (TFN)** [7]

TFN uses modality-specific encoders and computes an outer product interaction expansion over the three modality representations. The resulting tensor explicitly contains unimodal, bimodal, and trimodal interaction terms [7].

(6) **Low-Rank Multimodal Fusion (LMF)** [51]

LMF approximates the TFN interaction tensor using modality-specific low-rank factors. This substantially reduces the number of parameters and the computational cost of explicit tensor fusion [51].

(7) **Multimodal Transformer (MulT)** [52]

MulT uses directional pairwise cross-modal attention to transfer information between unaligned modality sequences. The attended representations are combined for prediction [52].

(8) **Quantum-Inspired Multimodal Fusion Network (QMF)** [53]

QMF embeds multimodal word representations in a complex-valued space. It constructs local context states using global weighting and local mixing. In its measurement-inspired step, multimodal observations are applied to each context state, and the rows of the resulting probability matrix are max-pooled for prediction.

(9) **Adaptive Language-Guided Multimodal Transformer (ALMT)** [54]

Table 2 summarizes the fusion strategies, principal advantages, and limitations of the reproduced baseline models and QRSAN. No single fusion strategy is optimal across all experimental settings. Recurrent fusion models are comparatively simple but provide limited explicit modelling of high-order interactions. Tensor-based methods capture richer interactions at a higher computational cost, whereas attention-based methods improve cross-modal alignment but generally require more parameters and training resources.

(10) **MEGAKANs** [55]

MEGAKANs combines global channel-spatial attention with Kolmogorov–Arnold Networks. The attention module adaptively emphasizes salient emotional information across channel and spatial dimensions, whereas KAN-based mid-level fusion captures high-order nonlinear dependencies among textual, acoustic, and visual modalities for sentiment prediction.

(11) **Multimodal Sentiment Analysis Based on Multi-Scale Feature Extraction and Multi-Task Learning (M3SA)** [56]

M3SA extracts representations from multiple hidden layers and integrates them through channel attention to capture multi-scale modality features. It then uses key-modality-guided fusion to emphasize the dominant modality and model its relationships with the remaining modalities. Multi-task learning is further used to improve feature representations and sentiment prediction.

EF-LSTM and LF-LSTM provide computationally simple baselines but offer limited explicit interaction modelling. TFN captures complete high-order interactions at a high computational cost, whereas LMF improves efficiency through low-rank approximation. MulT is suited to unaligned sequences but requires multiple cross-modal attention modules. QMF and QRSAN both employ quantum-inspired representations; however, QRSAN additionally uses normalized modality matrices, a TFN-derived interaction expansion, and real-valued residual self-attention. These components increase representational flexibility but also increase computational and memory requirements. ALMT uses multi-scale language features to guide adaptive hyper-modality learning and can suppress irrelevant or conflicting acoustic and visual information. Its reliance on language as the dominant modality may, however, limit adaptability when non-verbal cues are more informative. M3SA combines multi-scale feature extraction, key-modality-guided fusion, and multi-task learning to capture hierarchical representations and unequal modality contributions. Its performance may depend on accurate identification of the key modality and careful balancing of multiple training objectives. MEGAKANs integrates global channel-spatial attention with Kolmogorov–Arnold Networks to highlight salient emotional cues and capture high-order nonlinear intermodal dependencies. Nevertheless, its KAN-based components increase optimization and implementation complexity, and its generalizability requires further evaluation across multimodal sentiment datasets.

All baseline results in Table 3 and Table 4 were obtained by running the PyTorch implementations released with the codebase rather than copied from the original papers. EF-LSTM, LF-LSTM, MARN, MFN, TFN, LMF, MulT, QMF, ALMT, MEGAKANs, M3SA, and QRSAN used identical preprocessed features, train-validation-test splits, labels, and evaluation scripts. Models were selected on validation performance. For QRSAN, the validation-set grid-search protocol and search space are described in Section 3.5. For reproduced baseline models, common hyperparameters were selected on the validation set, whereas architecture-specific settings followed the corresponding released implementations. For the pre-specified paired statistical analyses, the reproduced comparator was ALMT for CMU-MOSEI, MEGAKANs for CMU-MOSI, and EF-LSTM for IEMOCAP. Before resampling, one eligible reproduced comparator was fixed for each dataset from baselines evaluated under the same train-validation-test split, preprocessing pipeline, labels, and evaluation scripts as QRSAN. The selected comparators were the strongest eligible baselines under the pre-defined primary metric for their respective datasets, and matched per-instance predictions were retained for paired inference. For each dataset, matched per-instance test predictions from five matched random-seed runs were retained for QRSAN and the corresponding comparator. All paired analyses used identical data splits, preprocessing, labels, and evaluation scripts within each dataset.

### 4.3. Main Experiment Results

The QRSAN hyperparameters were selected by validation-set grid search, as described in Section 3.5. The search covered batch size, learning rate, gradient-norm clipping threshold, training epochs, random seed, hidden dimension, and the number of projection kernels. The test set was used only for final evaluation. Each model was trained independently five times with different random seeds using identical data splits, feature representations, and evaluation scripts. Table 3 and Table 4 report mean performance across the five runs. For each dataset-metric comparison, the reported effect was the performance difference between QRSAN and the pre-specified comparator on matched test predictions. We calculated 95% confidence intervals using 10,000 paired bootstrap resamples of test instances. We tested the null hypothesis of no difference using 10,000 paired prediction-swap permutations, in which the paired model predictions were exchanged independently with probability 0.5 for each test instance. The three retained metrics across the three datasets yielded nine planned comparisons; Holm’s step-down adjustment was applied jointly across all nine *p* values. The full baseline tables remain descriptive because matched per-instance predictions were not retained for every reproduced baseline.

ACC denotes classification accuracy, namely binary accuracy for CMU-MOSI and CMU-MOSEI and micro-averaged label-wise accuracy across four one-vs-rest binary emotion-label tasks for IEMOCAP. ACC_5 and ACC_7 denote five-class and seven-class accuracy after discretizing sentiment-intensity labels. Binary_F1 is the F1 score for binary sentiment classification, and Recall is the corresponding binary recall. MAE is the mean absolute error between predicted and reference sentiment scores, whereas r is the Pearson correlation coefficient. Higher values are better for ACC, ACC_5, ACC_7, Binary_F1, Recall, and r; lower values are better for MAE. For the retained-prediction analyses, balanced accuracy is the mean recall across classes, and macro-F1 is the unweighted mean of class-specific F1 scores. For IEMOCAP, these metrics are macro-averaged across the four one-vs-rest binary emotion-label tasks.Table 5 summarizes the class-imbalance robustness results based on the retained test predictions.

Table 3 summarizes classification performance using the evaluation protocol described above. QRSAN was competitive across the three datasets, although it did not achieve the highest value for every metric. On CMU-MOSEI, QRSAN achieved 77.53 ± 1.70% ACC and 77.84 ± 1.42% Binary_F1. Its ACC matched that of MulT and was 0.57 percentage points below that of ALMT, whereas its Binary_F1 was 0.03 percentage points below that of MEGAKANs. QRSAN achieved the second-highest Recall, 89.52 ± 1.69%, trailing QMF by 1.10 percentage points. Its ACC_5 and ACC_7 were below the best values obtained by MulT.

On CMU-MOSI, QRSAN achieved the highest ACC and Binary_F1 among the compared models, reaching 76.10 ± 1.10% and 76.09 ± 1.12%, respectively. Its Recall was 88.50 ± 1.28%, 0.46 percentage points below the highest value achieved by LF-LSTM. QRSAN achieved 33.96 ± 2.08% ACC_5 and 30.89 ± 1.07% ACC_7, both below the best values reported by LF-LSTM and MulT, respectively. On IEMOCAP, QRSAN achieved 79.94 ± 0.94% ACC, 1.68 percentage points below EF-LSTM. These results support competitive performance under the adopted protocol, particularly for binary sentiment classification, but do not establish universal state-of-the-art performance or robustness to all forms of label imbalance.

The regression results in Table 4 further indicate that QRSAN achieved performance comparable to that of the evaluated baselines. On CMU-MOSEI, QRSAN achieved an MAE of 0.6368 ± 0.0070, which was 0.0183 above the best result from MEGAKANs. On CMU-MOSI, it achieved an MAE of 1.0101 ± 0.0353, ranking third and remaining close to the best value of 0.9986 obtained by MulT. These results indicate comparable continuous sentiment-prediction performance under the stated evaluation protocol.

#### Robustness to Class Imbalance

To rule out trivial performance explanations, we conducted a post hoc class-imbalance robustness analysis using five retained QRSAN test-prediction runs. Majority-class labels were determined from the training split only, and uniform-random baselines were estimated from 1000 deterministic draws. Each method was evaluated on the same retained test instances within a dataset: 686 for CMU-MOSI, 4643 for CMU-MOSEI, and 938 for IEMOCAP.

The canonical QRSAN accuracies are 77.53 ± 1.70% on CMU-MOSEI, 76.10 ± 1.10% on CMU-MOSI, and 79.94 ± 0.94% on IEMOCAP (Table 3). As shown in Table 5, the retained-prediction analysis additionally gives 73.28 ± 1.20% balanced accuracy and 73.03 ± 1.63% macro-F1 on CMU-MOSEI; QRSAN exceeded both trivial references on these metrics. On CMU-MOSI, QRSAN likewise exceeded the majority and uniform-random references on the retained balanced-accuracy and macro-F1 measures. On IEMOCAP, it exceeded both references for these measures despite the high nominal accuracy of the all-negative majority classifier.

Table 6 shows that QRSAN significantly exceeded ALMT on CMU-MOSEI for ACC, balanced accuracy, and macro-F1. Its differences from MEGAKANs on CMU-MOSI were not statistically supported and are therefore described as competitive. For IEMOCAP, the retained one-vs-rest emotion-label evaluation shows that QRSAN was significantly lower than EF-LSTM on all three retained-prediction metrics.

### 4.4. Class-Imbalance Analysis

To characterize label imbalance, for CMU-MOSI and CMU-MOSEI binary evaluation, zero-valued labels are retained and assigned to the non-negative class rather than excluded. IEMOCAP is evaluated through four independent one-vs-rest binary emotion-label tasks. The class distributions used in the reported analyses are calculated directly from the corresponding label arrays.

**Figure 4:**
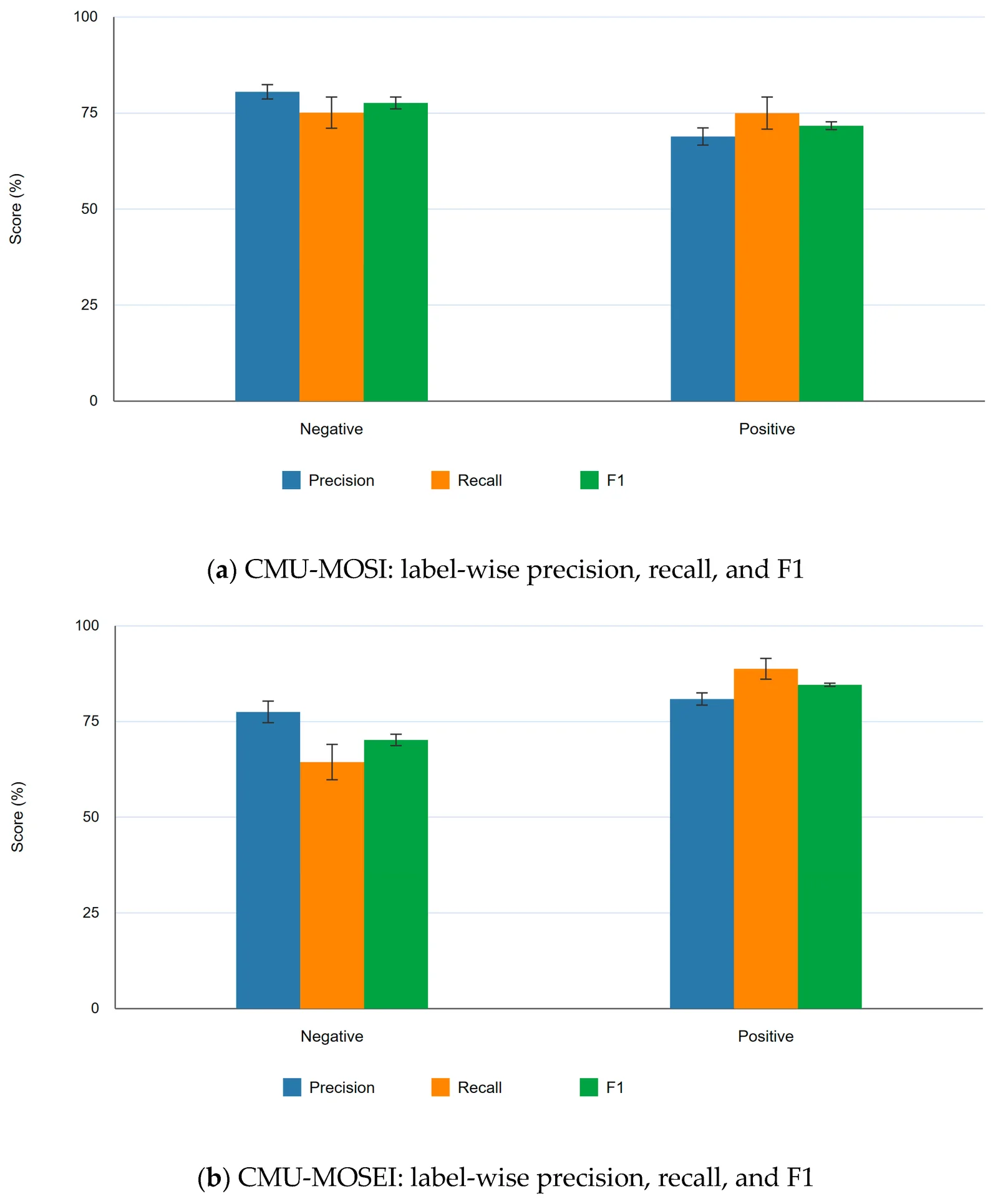

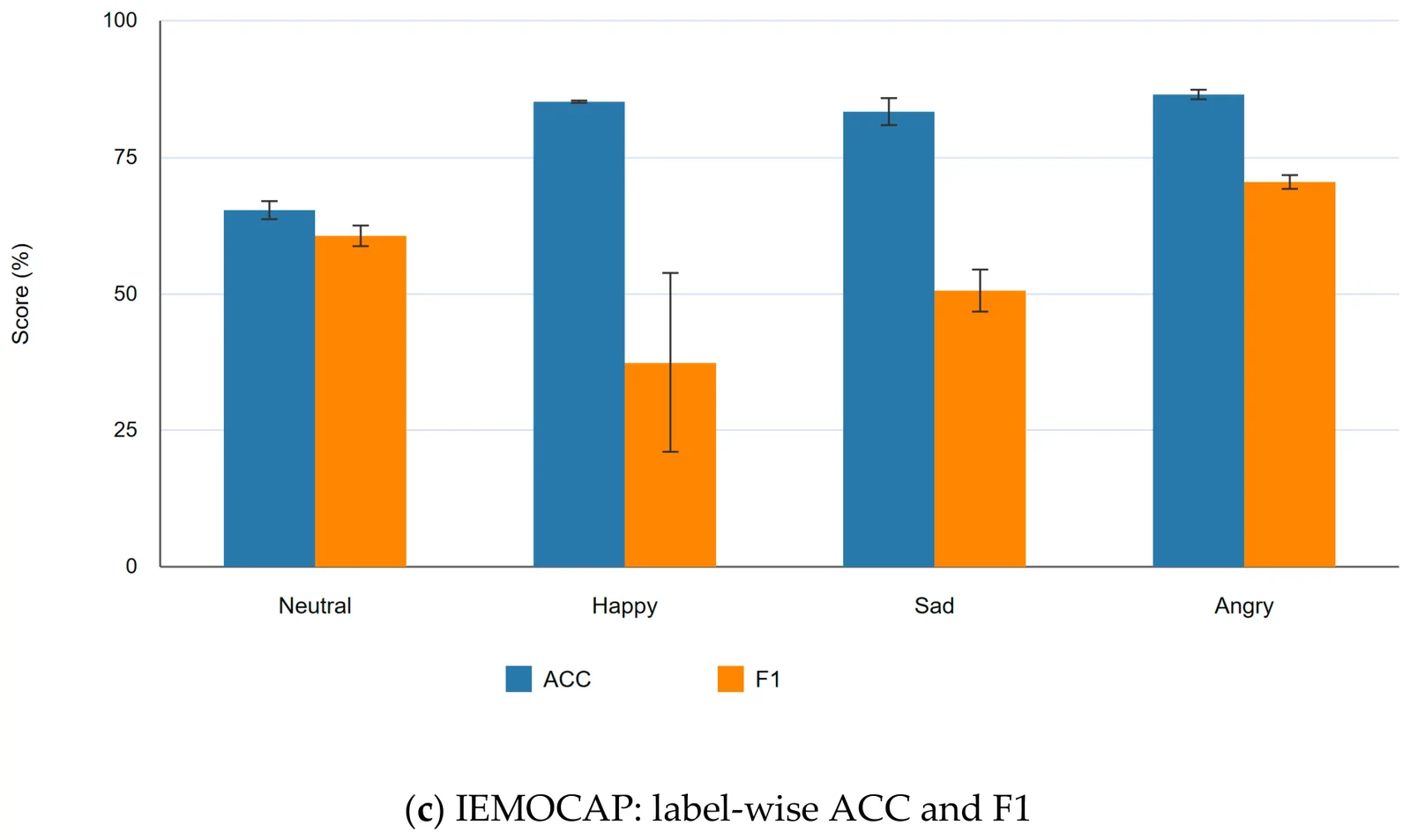
Label-wise performance under the label distributions of (**a**) CMU-MOSI, (**b**) CMU-MOSEI, and (**c**) IEMOCAP. The vertical axis reports score (%).

Figure 4 complements aggregate accuracy with label-wise results. For CMU-MOSI and CMU-MOSEI, it reports precision, recall, and F1 for the positive and negative classes. For IEMOCAP, it reports label-wise ACC and F1 for neutral, happy, sad, and angry. Performance varied across datasets and labels. In particular, the lower and more variable F1 for the happy class indicates that minority-emotion recognition remains challenging. Label-wise recall and F1 should therefore be interpreted alongside overall accuracy, especially for imbalanced labels.

Multimodal representations can integrate semantic, facial, and prosodic evidence when one cue is weak. However, dedicated imbalance-handling methods and evaluation under stronger distribution shifts remain necessary.

### 4.5. Ablation Experiments

**Figure 5:**
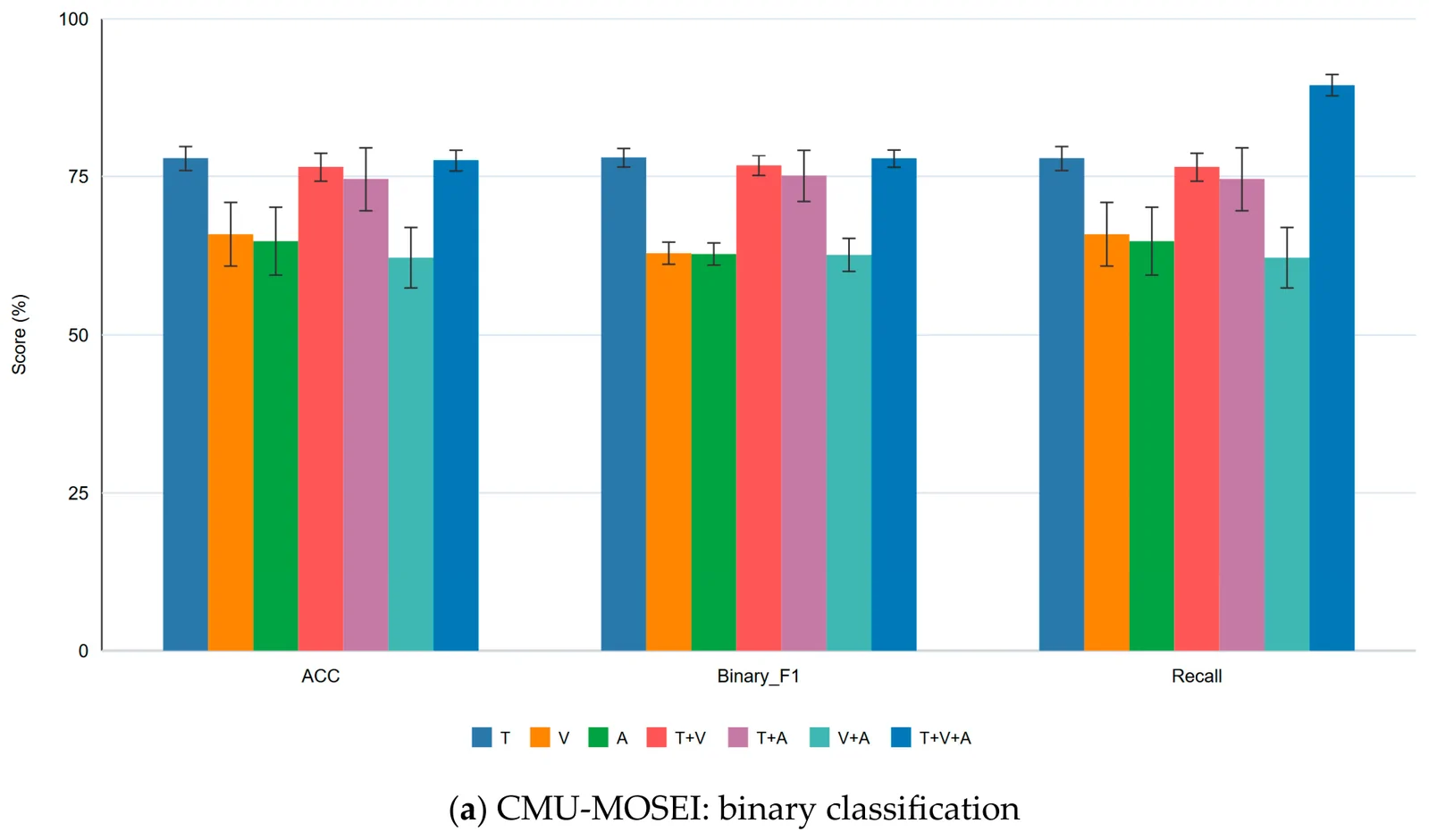

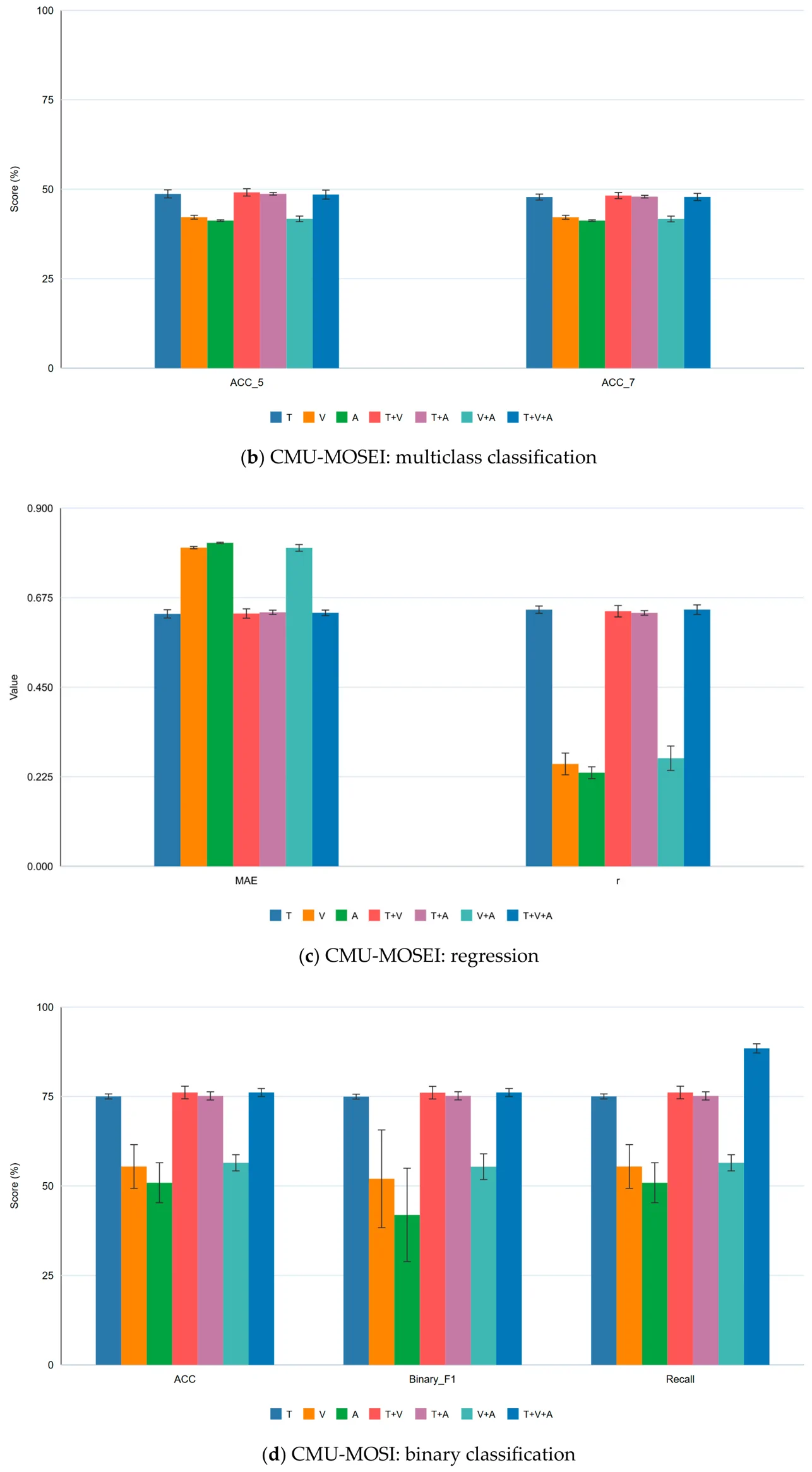

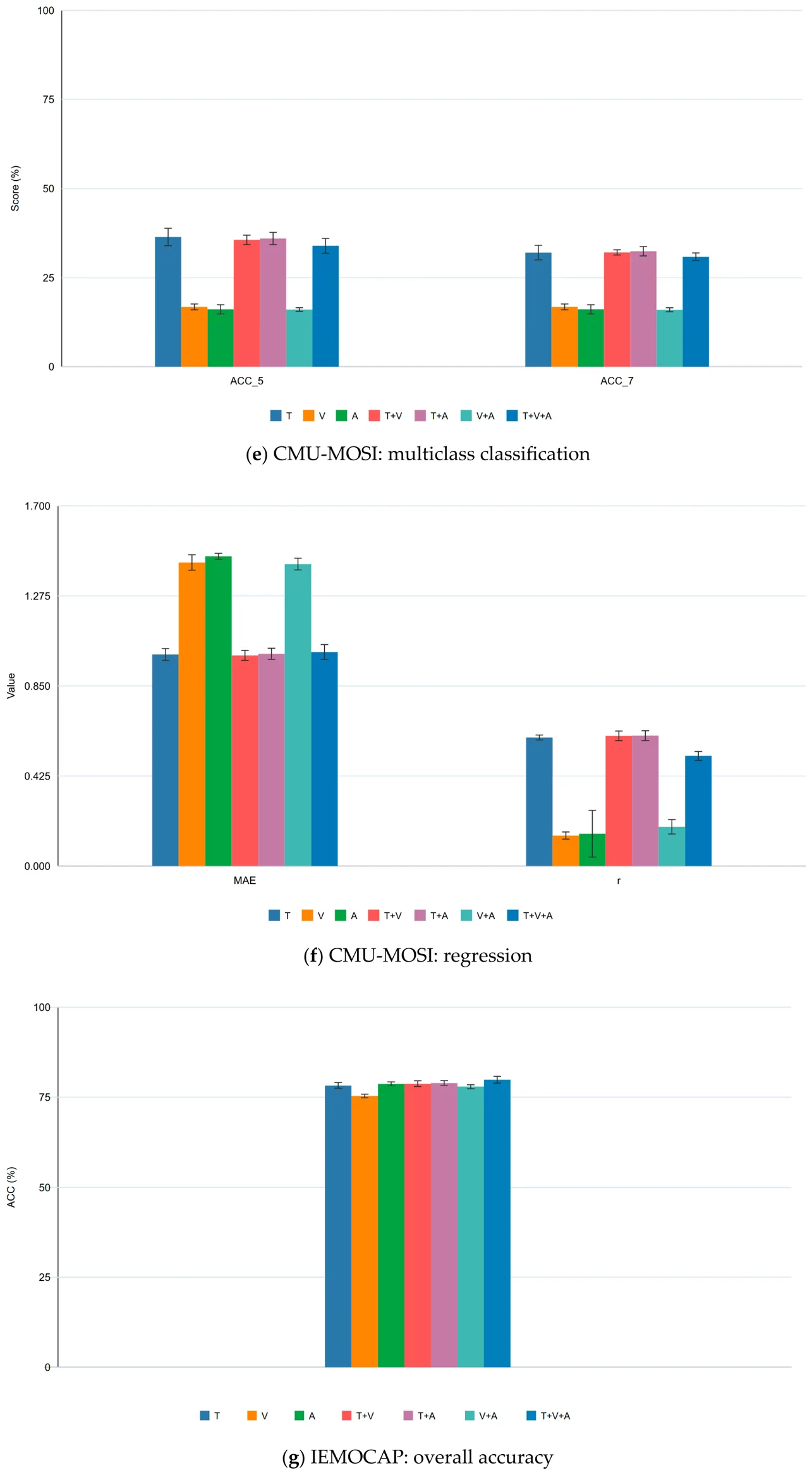

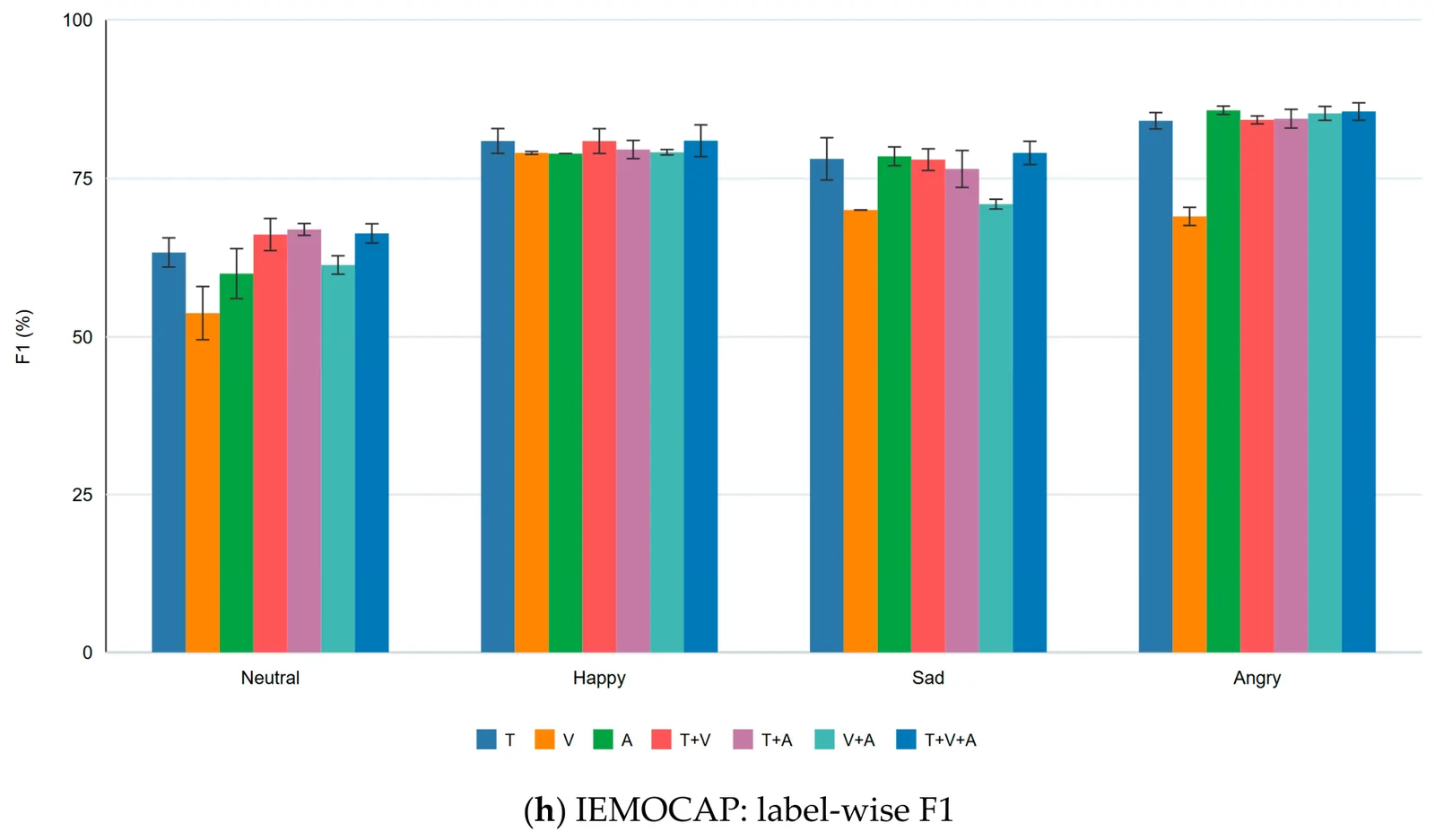
Performance of text (T), visual (V), acoustic (A), bimodal, and trimodal input combinations on CMU-MOSEI, CMU-MOSI, and IEMOCAP. Each bar shows the mean over five independent runs, and error bars denote the standard deviation. Classification metrics are reported as percentages; higher values are better. For regression, lower MAE and higher Pearson correlation coefficient r indicate better performance.

Modal ablation experiments. Figure 5 reports the mean and standard deviation over five independent runs for unimodal, bimodal, and trimodal QRSAN configurations. These experiments evaluate the contributions of textual, visual, and acoustic inputs on CMU-MOSI, CMU-MOSEI, and IEMOCAP. Results are reported separately for classification, regression, overall accuracy, and label-wise F1, where applicable.

Figure 5 shows that modality contributions depended on both dataset and metric. On CMU-MOSEI, the trimodal configuration performed strongly, particularly for Recall, although textual and text-visual configurations remained competitive on several classification metrics. On CMU-MOSI, trimodal fusion yielded competitive ACC and Binary_F1, whereas textual or bimodal configurations performed better on some fine-grained metrics. On IEMOCAP, trimodal fusion achieved competitive overall accuracy, but differences among several multimodal settings were small. These findings indicate that multimodal fusion can integrate complementary evidence but does not guarantee the best result for every metric or dataset.

Furthermore, we evaluated component-removed variants using the same trimodal input, data splits, evaluation script, and seed set. The results for the full QRSAN configuration were taken from the main experiments, whereas the component-removed variants were evaluated in separate ablation experiments. The results provide descriptive sensitivity estimates for the contribution of individual components. The variants remove complex encoding, learned phase, mixture weighting, TFN-derived interaction expansion, self-attention, or residual addition; a real-and-imaginary concatenation followed by an MLP serves as a classical feature-mapping comparator. Table 7 reports the resulting ACC values. These ablations test sensitivity to individual design choices and do not establish that any single component improves every dataset.

The controlled results show heterogeneous effects. For example, removing learned phase reduced ACC on CMU-MOSEI and IEMOCAP relative to the full configuration, whereas several simplified variants matched or exceeded the full configuration on CMU-MOSI. Accordingly, the evidence supports dataset-dependent utility of the proposed components rather than a universal component-wise advantage.

The component-removed variants retain their respective five-seed ablation results and should be interpreted with their uncertainty estimates; no component-wise statistical-significance claim is made here.

We examined the dependence on the final pooling and classifier head using five runs (Table 8). The alternative heads were evaluated in separate controlled sensitivity runs. Mean pooling achieved the highest ACC on CMU-MOSEI, whereas weighted pooling with a linear head achieved the highest ACC on CMU-MOSI and IEMOCAP. We retain max pooling as the pre-specified default QRSAN head, while recognizing that the preferred aggregation strategy is dataset-dependent.

For classification, the real-valued class-logit vector is normalized by Softmax and optimized using cross-entropy loss. For regression on CMU-MOSI and CMU-MOSEI, the same feature extractor and projection-score mapping are followed by a separate scalar prediction head. The scalar output is trained against the continuous sentiment score using MAE loss. MAE and the Pearson correlation coefficient r are calculated from test-set predictions, and validation-set MAE is used for model selection.

**Projection-kernel redundancy analysis.** To assess redundancy, we computed the absolute complex cosine similarity, namely the normalized magnitude of the Hermitian inner product between two kernels, for all 190 kernel pairs in each of the five saved main-QRSAN checkpoints per dataset. The results are summarized in Table 9. The mean pairwise similarity was 0.313 ± 0.040 on CMU-MOSEI, 0.474 ± 0.056 on CMU-MOSI, and 0.395 ± 0.041 on IEMOCAP. No CMU-MOSEI pairs exceeded 0.90 similarity, whereas 21 of 950 CMU-MOSI pairs and 6 of 950 IEMOCAP pairs did so across the five runs. These results do not indicate universal collapse of the kernels, but the high-similarity pairs show that strict non-redundancy is not guaranteed.

Basis-permutation sensitivity analysis. We evaluated each fixed seed-77 QRSAN checkpoint under its identity ordering and ten random permutations of the $D_{aug}=1331$ learned augmented tensor-product coordinates. A permutation matrix is unitary; thus, this experiment probes a restricted unitary transformation class. The permutations act on the augmented representation of dimension $D_{aug}=1331$, rather than on the unaugmented core dimension $D_{core}=1000$. Table 10 shows that random coordinate permutations changed the outputs and reduced the mean test accuracy, particularly on CMU-MOSI. The results therefore demonstrate coordinate-basis sensitivity rather than invariance. They quantify perturbations of one trained checkpoint on a fixed test set, not independent training repetitions or sensitivity to arbitrary dense unitary transformations.

### 4.6. Computational Complexity and Empirical Efficiency

We profiled QRSAN, TFN, LMF, QMF, and MulT across five independently completed runs per dataset. The measured computational profiles are summarized in Table 11. All measurements used a batch size of 32 on an NVIDIA GeForce RTX 3090 GPU. Trainable parameter counts were obtained directly from the trained models. Training time denotes the archived wall-clock duration of the completed training phase, test inference time denotes forward-pass latency per test example excluding data loading, and peak GPU memory denotes the maximum recorded allocation during each run.

The measured profile reveals a dataset-dependent efficiency trade-off. QRSAN uses fewer peak GPU-memory resources than QMF and MulT on all three datasets, but it is slower than LMF and TFN. Its training time is lower than that of QMF and MulT for all three datasets, whereas its test inference latency is lower than QMF and MulT on CMU-MOSEI and CMU-MOSI and lower than QMF on IEMOCAP. These measurements do not establish a universal efficiency advantage because the models differ in architecture and parameterization.

**Figure 6:**
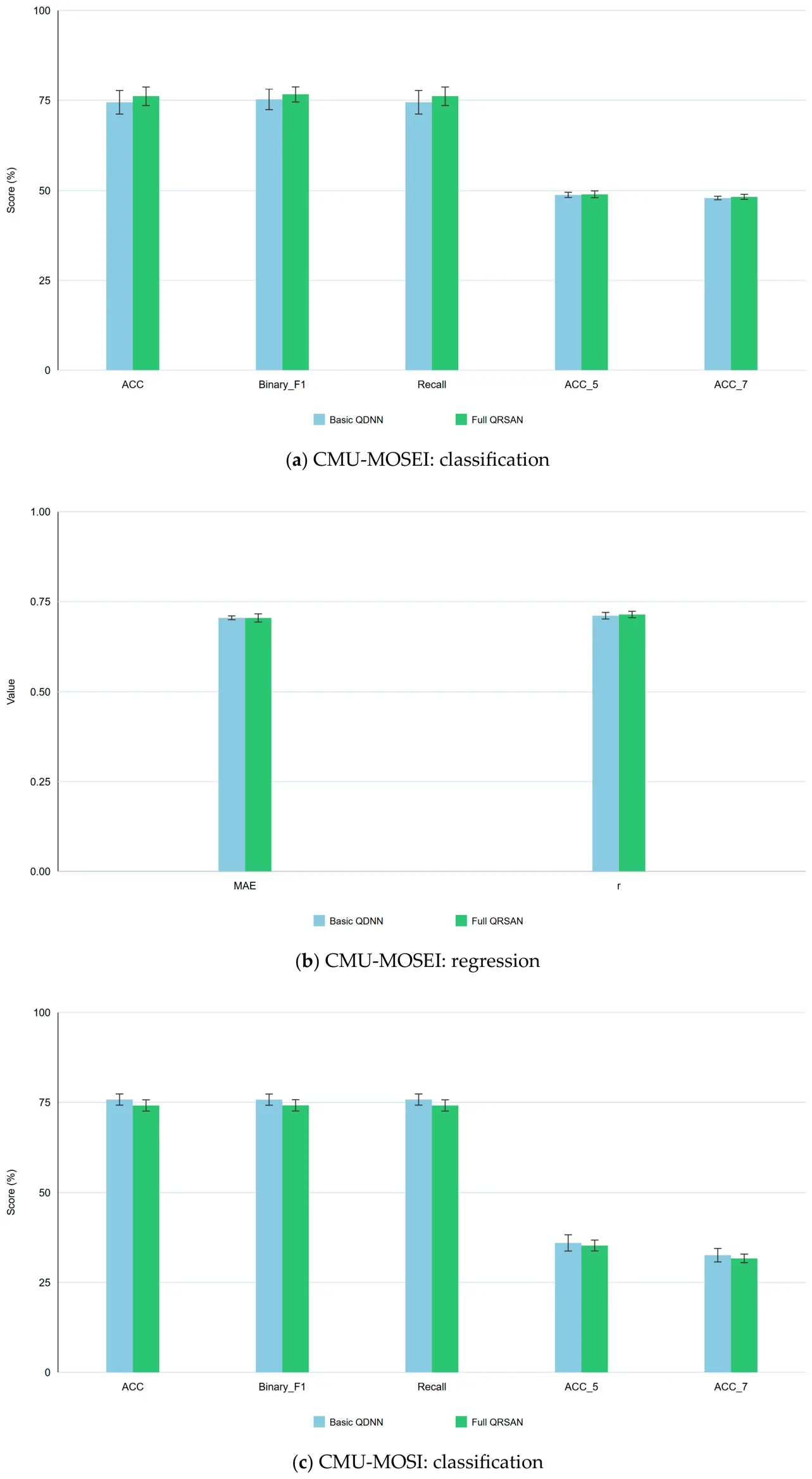

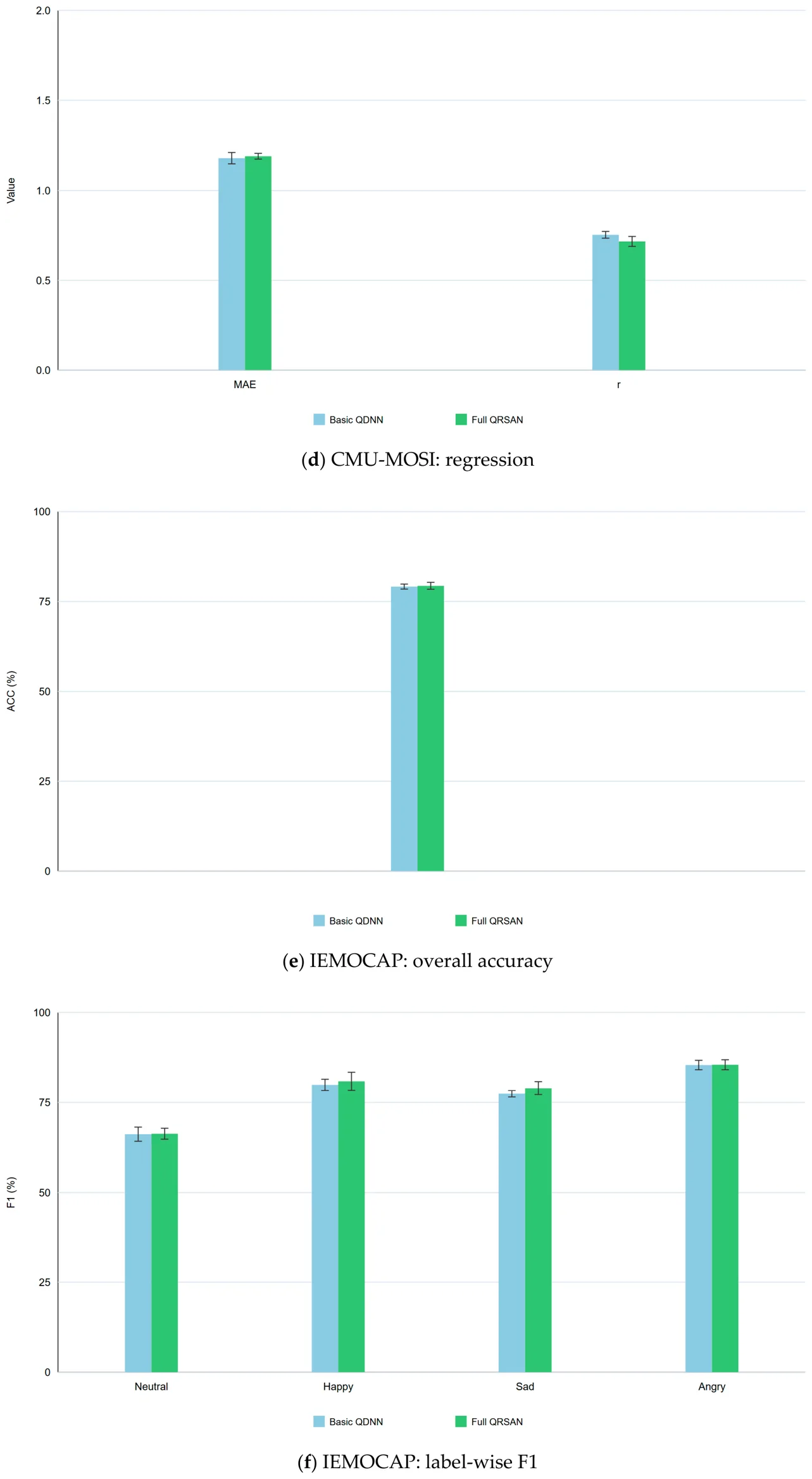
Comparison between Basic QDNN and Full QRSAN with the proposed residual self-attention module on CMU-MOSEI, CMU-MOSI, and IEMOCAP. Each bar shows the mean over five independent runs, and error bars denote the standard deviation. Higher values are better for classification metrics and Pearson correlation coefficient r, whereas lower MAE is better.

Figure 6 compares Basic QDNN with Full QRSAN incorporating the proposed residual self-attention module on CMU-MOSEI, CMU-MOSI, and IEMOCAP. The results are presented as means and standard deviations over five independent runs.

## 5. Conclusions and Future Work

We presented QRSAN, a quantum-inspired residual self-attention network for multimodal sentiment analysis. Using the common evaluation protocol, QRSAN achieved 76.10 ± 1.10% ACC and 1.0101 ± 0.0353 MAE on CMU-MOSI, 77.53 ± 1.70% ACC and 0.6368 ± 0.0070 MAE on CMU-MOSEI, and 79.94 ± 0.94% ACC on IEMOCAP. Among the models evaluated using the stated protocol, it achieved the highest numerical mean ACC and Binary_F1 on CMU-MOSI and remained competitive on CMU-MOSEI and IEMOCAP. The label-wise and sensitivity analyses indicate that the complete architecture is a competitive configuration under the evaluated settings; however, the ablation results are dataset-dependent and do not establish a universal advantage for every individual component. These results do not establish universal state-of-the-art performance. Hardware specifications and five-run peak GPU-memory measurements are reported; additional profiling across broader hardware and batch-size settings would further characterize scalability.

QRSAN runs entirely on classical hardware. Its complex-valued vectors, positive semidefinite modality matrices, TFN-derived interaction expansion, and projection-score mapping are representation-learning tools rather than physical quantum operations. Softmax is applied only to real-valued similarity or class-score matrices, and the residual-attention output is treated as a classical complex-valued feature matrix. Key limitations include the computational cost of interaction expansion, reliance on pre-extracted aligned features, and evaluation on three corpora. Future work will examine alternative classical projection-score mappings with stronger mathematical consistency, low-rank or sparse interaction expansions, and additional corpora with missing modalities, label imbalance, and distribution shifts. Hybrid quantum-classical implementations may also be explored where physically meaningful formulations are available.

## Figures and Tables

**Table 1:** Dataset details.

Dataset	Split	Number of Samples	Total	Labels
CMU-MOSI	Train	1284	2199	2, 5, 7
	Validation	229		
	Test	686		
CMU-MOSEI	Train	16,265	22,777	2, 5, 7
	Validation	1869		
	Test	4643		
IEMOCAP	Train	2717	4453	4
	Validation	798		
	Test	938		

**Table 2:** Qualitative comparison of the baseline models and QRSAN.

Model	Fusion Strategy	Main Advantages	Main Limitations
EF-LSTM	Early concatenation followed by an LSTM	Simple architecture, relatively low implementation cost, and joint temporal modeling after feature concatenation	Direct concatenation does not explicitly model high-order cross-modal interactions and generally assumes adequately aligned inputs
LF-LSTM	Separate modality-specific LSTMs followed by late concatenation	Preserves modality-specific temporal representations and is less affected by heterogeneous input dimensions	Cross-modal interaction is introduced only after unimodal encoding, which limits fine-grained information exchange
MARN	Multi-attention recurrent fusion	Captures temporal dependencies and dynamically models interactions among modalities	Recurrent multi-attention increases architectural complexity and can be computationally expensive for long sequences
MFN	Modality-specific LSTMs with an attention-based memory system	Models view-specific and cross-view temporal dynamics through a shared memory mechanism	The memory architecture contains multiple recurrent components and may be difficult to optimize
TFN	Outer product tensor fusion	Explicitly preserves unimodal, bimodal, and trimodal interaction terms	The tensor dimension grows rapidly with modality dimensions, resulting in high memory and computational costs
LMF	Low-rank tensor factorization	Considerably reduces the computational and memory costs of full tensor fusion	The low-rank constraint may discard informative high-order interactions, and performance can depend on the selected rank
MulT	Directional pairwise cross-modal Transformer attention	Effectively models unaligned multimodal sequences and long-range cross-modal dependencies	Pairwise Transformer modules introduce substantial parameter and training costs and do not directly represent complete trimodal interactions
QMF	Quantum-inspired complex-valued representation and measurement-based fusion	Provides a structured complex-valued representation for modeling multimodal correlations	Its interpretation depends on quantum-inspired assumptions, and it does not explicitly combine TFN-derived lower- and higher-order interactions with residual self-attention
ALMT	Multi-scale language-guided Adaptive Hyper-modality Learning (AHL), followed by fusion of the learned audio-visual hyper-modality representation with language features	Suppresses sentiment-irrelevant and conflicting information in auxiliary modalities and produces complementary joint representations	Assumes that language is the dominant and reliable modality; performance may degrade when textual cues are ambiguous or when audio/visual information is more informative, while the multi-stage Transformer architecture adds computational overhead
MEGAKANs	Global channel-spatial attention with KAN-based mid-level fusion for learning nonlinear interactions among textual, acoustic, and visual features	Captures high-order nonlinear intermodal dependencies, highlights salient emotional patterns across channel and spatial dimensions, and provides greater functional interpretability than conventional MLP fusion	KAN and global attention operations increase optimization and implementation complexity; validation is currently limited mainly to the CMU-MOSI dataset, leaving cross-dataset generalizability insufficiently established
M3SA	Multi-scale feature extraction, key-modality-guided fusion, and multi-task learning	Captures hierarchical modality representations and models unequal modality contributions through key-modality guidance	Performance depends on reliable key-modality identification and careful balancing of multiple training objectives
QRSAN	Normalized complex-valued encoding, TFN-derived interaction expansion, and residual self-attention	Explicitly models different orders of cross-modal interaction while adaptively emphasizing sentiment-relevant features	The Kronecker-product expansion and complex-valued matrix operations increase memory use, computational cost, and implementation complexity

**Table 3:** Classification experiment performance. (Values are averaged over five independent runs. For QRSAN, mean ± standard deviation is reported.) For IEMOCAP, label-wise ACC is the micro-averaged accuracy across the four one-vs-rest binary emotion-label decisions.

**Model**	**CMU-MOSEI**					
	**ACC ↑**	**ACC_5 ↑**	**ACC_7 ↑**	**Binary_F1 ↑**	**Recall ↑**	
EF-LSTM	76.59	47.78	47.02	76.72	86.16	
LF-LSTM	75.60	48.88	48.00	75.56	88.34	
MARN	74.91	49.81	48.78	75.52	88.83	
MFN	74.05	50.31	49.12	74.69	88.94	
TFN	75.10	50.55	49.42	75.26	88.42	
LMF	77.08	50.25	49.06	77.45	89.14	
MulT	77.53	**51.09**	**49.90**	77.86	87.59	
QMF	75.91	48.66	47.72	76.50	**90.62**	
ALMT	**78.10**	49.00	48.09	77.66	84.24	
MEGAKANs	77.66	50.52	49.38	**77.87**	88.38	
M3SA	74.47	44.44	44.17	74.68	84.34	
QRSAN	77.53 ± 1.70	48.49 ± 1.26	47.83 ± 1.00	77.84 ± 1.42	89.52 ± 1.69	
**Model**	**CMU-MOSI**					**IEMOCAP**
	**ACC ↑**	**ACC_5 ↑**	**ACC_7 ↑**	**Binary_F1 ↑**	**Recall ↑**	**Label-Wise** **ACC ↑**
EF-LSTM	73.81	35.30	31.40	73.86	86.99	**81.62**
LF-LSTM	73.33	**37.35**	**32.98**	73.32	**88.96**	80.80
MARN	73.84	35.18	31.22	73.71	87.61	81.25
MFN	74.79	36.13	32.26	74.70	88.14	71.70
TFN	72.59	36.10	31.70	72.53	86.78	80.27
LMF	72.83	34.11	30.54	72.78	86.92	80.23
MulT	74.52	36.52	32.41	74.49	85.65	79.39
QMF	73.18	35.95	32.08	73.23	85.44	78.71
ALMT	73.39	29.02	26.82	73.27	82.55	77.73
MEGAKANs	74.91	31.31	28.33	74.92	82.36	80.23
M3SA	69.85	31.07	28.78	69.73	78.53	77.62
QRSAN	**76.10 ± 1.10**	33.96 ± 2.08	30.89 ± 1.07	**76.09 ± 1.12**	88.50 ± 1.28	79.94 ± 0.94

↑ denotes that higher values indicate better performance, Bold values indicate the best-performing results.

**Table 4:** Regression experiment performance.

Model	MAE ↓	
	CMU-MOSEI	CMU-MOSI
EF-LSTM	0.6592	1.0422
LF-LSTM	0.6436	1.0060
MARN	0.6322	1.0163
MFN	0.6293	1.0119
TFN	0.6205	1.0529
LMF	0.6268	1.0373
MulT	0.6228	**0.9986**
QMF	0.6385	1.0315
ALMT	0.6479	1.1234
MEGAKANs	**0.6185**	1.0661
M3SA	0.7014	1.1544
QRSAN	0.6368 ± 0.0070	1.0101 ± 0.0353

↓ denotes that lower values indicate better performance. Bold values indicate the best-performing results.

**Table 5:** Robustness to class imbalance using saved test predictions. QRSAN balanced accuracy and macro-F1 are reported as mean ± standard deviation across five retained-prediction runs. Uniform-random results are reported as mean ± sample standard deviation over 1000 draws. For IEMOCAP, the accuracy column reports micro-averaged label-wise accuracy across the four one-vs-rest binary emotion-label decisions, whereas balanced accuracy and macro-F1 are macro-averaged across these four tasks.

Dataset	Method	Accuracy ↑ (%)	Balanced Accuracy ↑ (%)	Macro-F1 ↑ (%)
CMU-MOSI	QRSAN	76.10 ± 1.10	75.55 ± 0.83	75.66 ± 0.81
	Majority-class baseline	44.75 ± 0.00	50.00 ± 0.00	30.92 ± 0.00
	Uniform-random baseline	50.05 ± 1.93	50.05 ± 1.93	49.89 ± 1.92
CMU-MOSEI	QRSAN	77.53 ± 1.70	73.28 ± 1.20	73.03 ± 1.63
	Majority-class baseline	71.03 ± 0.00	50.00 ± 0.00	41.53 ± 0.00
	Uniform-random baseline	50.00 ± 0.74	50.00 ± 0.81	47.69 ± 0.72
IEMOCAP	QRSAN	79.94 ± 0.94	66.93 ± 1.75	67.20 ± 2.48
	Majority-class baseline	75.00 ± 0.00	50.00 ± 0.00	42.67 ± 0.00
	Uniform-random baseline	49.99 ± 0.81	49.97 ± 0.97	45.97 ± 0.76

↑ denotes that higher values indicate better performance.

**Table 6:** Paired statistical comparison with the pre-specified reproduced comparator. Differences are QRSAN minus comparator in percentage points. *p*-values are Holm-adjusted across all rows. Comparators were pre-specified as ALMT for CMU-MOSEI, MEGAKANs for CMU-MOSI, and EF-LSTM for IEMOCAP. Confidence intervals used 10,000 paired-bootstrap resamples; *p* values used 10,000 paired prediction-swap permutations and were Holm-adjusted across nine planned comparisons.

**Dataset**	**Metric**	**Comparator**	**Δ (pp)**	**95% CI (pp)**	
CMU-MOSEI	ACC	ALMT	2.28	1.48 to 3.09	
	Balanced acc.	ALMT	1.16	0.22 to 2.10	
	Macro-F1	ALMT	1.80	0.86 to 2.74	
CMU-MOSI	ACC	MEGAKANs	0.35	−1.90 to 2.60	
	Balanced acc.	MEGAKANs	0.47	−1.80 to 2.74	
	Macro-F1	MEGAKANs	0.57	−1.73 to 2.85	
IEMOCAP	ACC	EF-LSTM	−1.93	−3.19 to −0.68	
	Balanced acc.	EF-LSTM	−3.21	−4.99 to −1.43	
	Macro-F1	EF-LSTM	−3.30	−5.18 to −1.39	
**Dataset**	**Metric**	**Comparator**	**Adjusted *p***	**d_z**	**Conclusion**
CMU-MOSEI	ACC	ALMT	0.0008999	0.563	Superior
	Balanced acc.	ALMT	0.0032	1.282	Superior
	Macro-F1	ALMT	0.0008999	0.853	Superior
CMU-MOSI	ACC	MEGAKANs	1.0000	0.270	Competitive
	Balanced acc.	MEGAKANs	1.0000	0.308	Competitive
	Macro-F1	MEGAKANs	1.0000	0.361	Competitive
IEMOCAP	ACC	EF-LSTM	0.0008999	−2.543	Inferior
	Balanced acc.	EF-LSTM	0.0008999	−2.190	Inferior
	Macro-F1	EF-LSTM	0.0008999	−1.442	Inferior

**Table 7:** Controlled component ablations (ACC %, mean ± standard deviation over five runs).

Variant	CMU-MOSEI	CMU-MOSI	IEMOCAP
Full QRSAN	77.53 ± 1.70	76.10 ± 1.10	79.94 ± 0.94
Without complex encoding	77.02 ± 1.79	74.90 ± 0.90	79.85 ± 0.80
Without learned phase	74.56 ± 3.03	75.01 ± 0.81	79.32 ± 1.13
Without mixture weighting	77.01 ± 1.82	75.71 ± 1.35	79.59 ± 0.52
Without TFN expansion	76.73 ± 3.64	75.34 ± 1.83	79.12 ± 0.64
Without self-attention	76.59 ± 3.35	75.69 ± 0.88	79.79 ± 0.83
Without residual addition	77.08 ± 2.36	75.42 ± 0.87	79.21 ± 0.62
Real/imaginary concat. + MLP	77.31 ± 3.31	75.22 ± 1.05	79.52 ± 0.49

**Table 8:** Projection-head sensitivity (ACC %, mean ± standard deviation over five runs). The alternative-head rows are separate controlled sensitivity runs.

Projection Head	CMU-MOSEI	CMU-MOSI	IEMOCAP
Max pooling + MLP (full QRSAN)	77.53 ± 1.70	76.10 ± 1.10	79.94 ± 0.94
Mean pooling + MLP	78.14 ± 1.12	72.45 ± 0.56	78.82 ± 0.90
Attention pooling + MLP	77.88 ± 2.55	72.51 ± 1.99	79.00 ± 0.14
Weighted pooling + linear	77.39 ± 2.10	76.27 ± 0.96	80.03 ± 0.40
Weighted pooling + MLP	76.76 ± 0.96	75.22 ± 0.74	79.98 ± 1.11

**Table 9:** Projection-kernel redundancy analysis across five saved main-QRSAN checkpoints. Absolute complex cosine similarity was calculated for 190 within-checkpoint kernel pairs, yielding 950 pairs per dataset.

Dataset	Mean Absolute Complex Cosine Similarity	Pairs with Similarity ≥0.90 (of 950)
CMU-MOSEI	0.313 ± 0.040	0
CMU-MOSI	0.474 ± 0.056	21
IEMOCAP	0.395 ± 0.041	6

**Table 10:** Basis-permutation sensitivity of fixed seed-77 QRSAN checkpoints. Permuted values are mean ± standard deviation over ten random permutations of the same fixed test set. ΔACC is relative to the identity ordering. For IEMOCAP, ACC is micro-averaged label-wise accuracy. These are sensitivity perturbations, not independent training runs.

Dataset	Identity ACC (%)	Permuted ACC (%)	ΔACC (pp)	Mean Absolute Output Change
CMU-MOSEI	78.01	75.46 ± 2.83	−2.55 ± 2.83	0.528 ± 0.027
CMU-MOSI	76.38	43.67 ± 3.31	−32.71 ± 3.31	1.099 ± 0.033
IEMOCAP	79.10	68.64 ± 4.11	−10.47 ± 4.11	1.055 ± 0.018

**Table 11:** Measured computational profile of the reproduced models. Values are mean ± standard deviation across five completed runs per dataset. Lower values are preferable for training time, test inference latency, and peak GPU memory.

**Dataset**	**Model**	**Parameters (M)**	**Training Time** **(min/run)**
CMU-MOSEI	LMF	0.248	3.76 ± 0.72
	MulT	1.892	180.80 ± 25.52
	QMF	5.604	120.80 ± 9.36
	QRSAN	5.714	76.05 ± 4.92
	TFN	9.314	3.99 ± 0.74
CMU-MOSI	LMF	0.245	0.57 ± 0.11
	MulT	1.889	19.52 ± 3.27
	QMF	1.259	11.11 ± 1.01
	QRSAN	1.330	6.16 ± 1.34
	TFN	9.311	0.72 ± 0.14
IEMOCAP	LMF	0.252	0.68 ± 0.09
	MulT	1.894	41.62 ± 2.80
	QMF	1.026	12.20 ± 1.41
	QRSAN	1.095	5.72 ± 1.21
	TFN	9.315	0.83 ± 0.13
**Dataset**	**Model**	**Peak GPU Memory (MiB)**	**Test Inference (ms/example)**
CMU-MOSEI	LMF	88.0 ± 10.7	0.10 ± 0.02
	MulT	495.5 ± 0.0	11.20 ± 8.93
	QMF	2584.7 ± 0.0	4.43 ± 0.39
	QRSAN	127.3 ± 0.0	3.02 ± 0.48
	TFN	195.2 ± 24.2	0.12 ± 0.05
CMU-MOSI	LMF	64.6 ± 0.0	0.10 ± 0.05
	MulT	479.7 ± 4.7	30.65 ± 2.28
	QMF	2551.5 ± 2.2	7.52 ± 0.89
	QRSAN	94.0 ± 2.3	3.02 ± 0.52
	TFN	168.9 ± 0.0	0.13 ± 0.05
IEMOCAP	LMF	46.1 ± 0.0	0.14 ± 0.05
	MulT	173.5 ± 0.0	28.46 ± 4.67
	QMF	1045.5 ± 1.5	3.64 ± 0.60
	QRSAN	52.3 ± 1.9	1.14 ± 0.32
	TFN	164.7 ± 0.0	0.08 ± 0.03

## Data Availability

The datasets used in this study, including CMU-MOSI, CMU-MOSEI, and IEMOCAP, are publicly available from their respective official repositories. The implementation code for this study is publicly available at https://github.com/pipijiev12/Multi_modal_sentiment_analysis (accessed on 6 September 2026).
